# Hypercrosslinked Polymers as a Photocatalytic Platform for Visible‐Light‐Driven CO_2_ Photoreduction Using H_2_O

**DOI:** 10.1002/cssc.202002824

**Published:** 2021-01-22

**Authors:** Giulia E. M. Schukraft, Robert T. Woodward, Santosh Kumar, Michael Sachs, Salvador Eslava, Camille Petit

**Affiliations:** ^1^ Barrer Centre Department of Chemical Engineering South Kensington Campus Imperial College London London SW7 2AZ UK; ^2^ Department of Chemical Engineering Imperial College London London SW7 2AZ UK; ^3^ Department of Chemistry White City Campus Imperial College London London W12 0BZ UK; ^4^ Current address: Institute of Materials Chemistry and Research, Faculty of Chemistry University of Vienna Währinger Straße 42 1090 Vienna Austria

**Keywords:** carbon dioxide, polymers, photocatalysis, porous organic polymers, solar fuels

## Abstract

The design of robust, high‐performance photocatalysts is key for the success of solar fuel production by CO_2_ conversion. In this study, hypercrosslinked polymer (HCP) photocatalysts have been developed for the selective reduction of CO_2_ to CO, combining excellent CO_2_ sorption capacities, good general stabilities, and low production costs. HCPs are active photocatalysts in the visible light range, significantly outperforming the benchmark material, TiO_2_ P25, using only sacrificial H_2_O. It is hypothesized that superior H_2_O adsorption capacities facilitate access to photoactive sites, improving photocatalytic conversion rates when compared to sacrificial H_2_. These polymers are an intriguing set of organic photocatalysts, displaying no long‐range order or extended π‐conjugation. The as‐synthesized networks are the sole photocatalytic component, requiring no added cocatalyst doping or photosensitizer, representing a highly versatile and exciting platform for solar‐energy conversion.

## Graphical abstract

**This is how it fuels**: Hypercrosslinked polymers are an intriguing set of organic photocatalysts. They seemingly display no extended π‐conjugation and are able to photoreduce gaseous CO_2_ to CO using H_2_O under visible light.

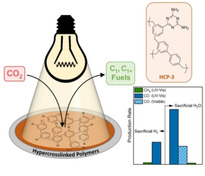

## Introduction

The ever‐increasing global energy demand requires a significant overhaul of current production processes if humanity is to address climate change. Carbon management and renewable energy must play a key role in our energy outlook, challenging researchers to reshape our energy portfolio. Research efforts are focused on the development of efficient carbon capture, utilization, and storage (CCUS) technologies, as well as the improvement of methods to harness renewable energy.^[1]^ The use of sunlight shows promise towards the building of a sustainable chemical industry. Solar fuels are synthetic fuels produced through conversion of solar energy into chemical energy, namely H_2_ from H_2_O, and C_1_ and C_1+_ chemicals from CO_2_. This conversion can be done by a variety of processes, including photochemical (often named artificial photosynthesis), thermochemical, and electrochemical reactions. However, overcoming the high thermodynamic and kinetic barriers to conversion is challenging, and so a catalyst is required to improve energy efficiency and, ultimately, render these processes viable.[^2^, ^3^]

Herein, we focus on a photochemical route to solar fuel production, namely photocatalysis, whose main advantage lies in the simplicity of its implementation. To date, ‘traditional’ semiconductors, such as metal oxides or sulfides, and transition metal complexes, such as TiO_2_, CdS, ZnO, WO_3_, Ru‐, Re‐ and Pd‐based complexes have received much attention as photocatalysts, owing to their ability to generate charge carriers under light irradiation.[^4^, ^5^, ^6^, ^7^] However, a lack of structural versatility and the notoriously difficult to tune frontier energy levels in inorganic materials often limit their performance, while the requirement of rare‐earth metals presents significant sustainability issues. Moreover, traditional semiconductors are often predominantly active at ultraviolet wavelengths, constituting only about 4 % of the solar spectrum. This has prompted great interest in the development of visible‐light‐active photocatalysts for improved efficiency.

The development of new classes of photoactive materials, including inorganic–organic hybrids, such as metal–organic frameworks (MOFs), or organic‐based materials, such as porous organic polymers, have emerged as promising alternatives to traditional photocatalysts.[^8^, ^9^, ^10^, ^11^, ^12^] The structural versatility of polymers enables photochemical tunability and, ultimately, optimization of photocatalytic performance. Owing to their general chemical inertness and nonmetallic nature, porous organic polymers are of particular interest in the design of new photocatalysts. Yang et al. reported triazine‐based conjugated microporous polymers (CMPs) for CO_2_ photoconversion to CO using visible light.^[13]^ The optical band gap of the materials was engineered by the inclusion of various electron‐withdrawing and electron‐donating groups. Yu et al. employed Pd‐catalyzed Sonogashira–Hagihara coupling to produce Eosin Y‐functionalized porous polymers, able to photoreduce CO_2_ to CO with 92 % selectivity, using visible light and sacrificial H_2_O.^[14]^ Liang et al. reported a rhenium‐metalated porous covalent organic framework (COF) for CO_2_ photoreduction to CO with a 97.8 % selectivity.^[15]^ More recently, Fu et al. synthesized a rhenium‐doped COF with high CO_2_ photoreduction rates in the presence of acetonitrile and sacrificial triethanolamine.^[12]^ Wisser et al. reported a three‐dimensional porous polymer made from organic photosensitizer heterogenized with rhodium active sites. They demonstrated the importance of directly tethering light harvesting components to catalytic sites, allowing efficient electronic energy transfer, enabling higher photo‐activity.^[16]^ Furthermore, a metal‐free COF produced using solvothermal condensation reactions was reported as a visible‐light‐driven photocatalyst for CO_2_ photoreduction in the presence of water.[^12^, ^17^] While demonstrating the potential of porous organic polymers for CO_2_ photoreduction, the synthesis of these photocatalysts generally required the use of rare‐earth metals, or specifically polymerizable monomeric units, presenting implementation barriers due to relatively high‐costs and poor sustainability.

Hypercrosslinked polymers (HCPs) represent a class of materials with excellent tunability and relatively low cost. HCPs are densely crosslinked amorphous networks that are produced by using simple Friedel–Crafts chemistry. Nonfunctional aromatic compounds (i. e., without specifically polymerizable groups) can be ‘knitted’ together using an external crosslinker, requiring only iron(III) chloride as catalyst.^[18]^ The employment of external crosslinkers means a large array of aromatic compounds can be considered as monomeric material, providing substantial scope for the design of HCPs. Owing to their low cost and chemical versatility, HCPs are being developed for many different applications such as gas separation and storage,[^19^, ^20^] solid‐state extraction,[^21^, ^22^] and catalysis.^[23]^ Recently, Wang et al. used an HCP‐TiO_2_‐graphene composite for the photoreduction of CO_2_, with the HCP component aiding CO_2_ adsorption and diffusion.^[24]^ The ability of HCPs alone to catalyze solar fuel production, however, remains unknown.

Herein, we present HCPs as a new class of photocatalyst capable of selectively reducing CO_2_ to CO. Photocatalytic conversion was achieved using only visible light in the presence of sacrificial H_2_O, without additional sacrificial agents or cocatalysts, significantly outperforming TiO_2_ P25 (7.5‐fold improvement). The influence of the reducing agent was investigated (i. e., H_2_ vs. H_2_O). Surprisingly, employing sacrificial H_2_O led to significant improvements in photoconversion rates. We hypothesize that the preferential adsorption of H_2_O concentrates the sacrificial agent at the HCP's surface, driving photocatalytic performance. Owing to their lack of requirement for precious‐metal catalysts, as well as their easily scaled chemistry, HCPs present an exciting platform for the further design and discovery of high‐performance organic photocatalysts.

## Results and Discussion

### Hypercrosslinked polymer synthesis and characterization

**Figure 1:**
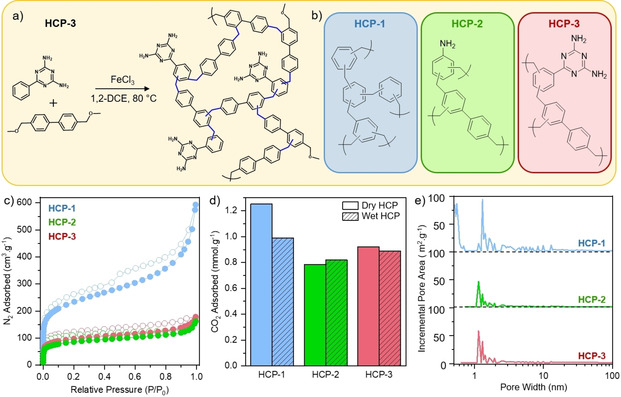
a) Reaction scheme for the production of HCP‐3 by Friedel–Crafts alkylation. b) Representative chemical structures of HCP repeat units. c) N_2_ adsorption isotherms at −196°C; filled symbols represent adsorption and empty symbols represent desorption. d) CO_2_ uptake at 1 bar and 25 °C for HCPs in both “dry” and “wet” states (i. e., HCPs exposed to humid atmospheres before measurement). e) Pore size distributions of HCPs calculated by using DFT methods.

We synthesized three HCPs of varied chemical structure – HCP‐1, HCP‐2, and HCP‐3 – by a Friedel–Crafts alkylation reaction using external crosslinkers to ‘knit’ together aromatic monomers. A general reaction scheme and representative HCP structures are shown in Figure 1a and 1b, respectively. HCP‐1 was produced through the crosslinking of benzene by using an aliphatic dimethoxymethane external crosslinker, one of the most widely‐studied HCPs in recent years.^[25]^ HCP‐2 is comprised of aniline crosslinked using the benzyl ether compound 4,4‐bis(methoxymethane)biphenyl, as the analogous polymer produced using dimethoxymethane was nonporous.^[26]^ Finally, HCP‐3 consisted of 2,4‐diamino‐6‐phenyl‐1,3,5‐triazine crosslinked using 4,4‐bis(methoxymethane)biphenyl. We chose the chemistries of HCP‐2 and HCP‐3 to try to improve the CO_2_ adsorption selectivity through the inclusion of amino groups. Photographic images of the networks (see the Supporting Information, Figure S2) reveal a color gradient from dark to light brown from HCP‐1 to HCP‐3. We successfully incorporated the aromatic monomers into the networks, as confirmed using a variety of characterization techniques. Solid‐state cross‐polarization magic angle spinning ^13^C NMR (ssNMR; Figure S3) of all polymers showed signals at around 139 and 129 ppm corresponding to quaternary aromatic carbons (C_Ar_) and aromatic C_Ar_−H.

Signals at around 36 ppm are assigned to newly formed methylene bridges, whereas weak signals at around 54 and 74 ppm correspond to unreacted methyl ethers O−CH_3_ and C_AR_C−O, respectively. The signal at 74 ppm is absent in HCP‐3, but a new signal emerges at around 102 ppm, suggesting this signal may be shifted downfield in this case. For HCP‐3, a signal at 166 ppm is assigned to triazine carbons and in HCP‐2 the C‐NH_2_ signal is expected at around 146 ppm and is therefore likely eclipsed by the substituted aromatic peak. Fourier‐transfer infrared spectroscopy (FTIR) showed distinct stretches for primary amines in HCP‐2 and HCP‐3, and the triazine tertiary amines in HCP‐3 (Figure S4). HCP‐2 and HCP‐3 contain 1.3 and 7.1 at% N, respectively, as determined by elemental analysis (Table S1), which may explain the lack of a signal in ssNMR for C‐NH_2_ in HCP‐2. These values correspond to final polymer compositions of roughly 1 : 4 and 1 : 3 monomer to crosslinker ratios for HCP‐2 and HCP‐3, respectively. Scanning electron micrographs revealed HCP‐1 and HCP‐3 as agglomerated spherical particles, whereas HCP‐2 shows a more fibrous structure (Figure S5). Thermogravimetric analysis demonstrated the high thermal stability of all HCPs, with decomposition temperatures of >300 °C in both N_2_ and air atmospheres (Figures S6 and S7). Char yields in N_2_ at 900 °C were >60 % in all HCPs, while complete degradation was observed in air at >550 °C. Powder X‐ray diffraction confirmed the amorphous nature of all HCP networks (Figure S8).

We used N_2_ sorption measurements at −196 °C to assess the porous nature of the networks. HCP‐1, HCP‐2, and HCP‐3 exhibited BET surface areas of 951, 311, and 357 m^2^/g, respectively (Figure 1c and Table S2). All HCPs displayed a combination of type I and type IV isotherms,^[27]^ with significant microporosity, as indicated by the steep N_2_ uptake at low relative pressures, as well as meso/macroporosity. Micropore volume was highest in HCP‐1 (0.46 cm^3^/g), dropping to 0.13 and 0.16 cm^3^/g for HCP‐2 and HCP‐3, respectively, reflecting trends seen in the polymer's BET surface areas. Figure 1e shows a multimodal pore size distribution for all networks, predominantly concentrated in the micropore region. Pores of around 0.5 nm in size contribute noticeably to HCP‐1’s surface area, whereas both HCP‐2 and HCP‐3 do not show any significant area derived from pores smaller than 1 nm in diameter (the first data point collected started above roughly 0.6 nm for HCP‐2 and HCP‐3, as no adsorption was detected below this range). To assess the CO_2_ uptake ability of HCPs, we collected adsorption isotherms at 25 °C up to 1 bar (full isotherms are shown in Figure S9). Although the HCPs followed the expected trend, whereby higher surface area polymers adsorbed more CO_2_, the CO_2_ capacities did not reflect the large differences in surface areas (Table S2). The presence of the amino groups in HCP‐2 and HCP‐3, which are known to impart CO_2_ selectivity to hypercrosslinked polymers,^[28]^ increased uptake density per unit of surface area due to more attractive interactions with the adsorbate CO_2_.[^29^, ^30^]

The presence of adsorbed water was shown to impede CO_2_ uptake in polar HCPs, due to competitive adsorption.^[31]^ Therefore, we investigated the effect of H_2_O and CO_2_ co‐adsorption, since we used sacrificial H_2_O vapor in CO_2_ photoreduction (see below). We exposed the samples to humid air (>99 % humidity) for at least 48 h before collecting CO_2_ adsorption isotherms at 25 °C up to 1 bar. Crucially, samples were not degassed prior to CO_2_ adsorption measurements (i. e., adsorbed H_2_O was not fully removed, see the Supporting Information for details). The CO_2_ sorption capacities at 1 bar are given in Figure 1d. The full isotherms of both degassed, “dry”, and humidity‐exposed, “wet”, HCPs are shown in Figure S9. Wet HCP‐1 showed a 21 % decrease in CO_2_ capacity in comparison to its dry equivalent, while the amine‐containing HCP‐2 and HCP‐3 showed negligible difference in CO_2_ uptake between the wet and dry networks. These uptake capacities demonstrate that CO_2_ can still adsorb in the presence of water. To further investigate the impact of water, we measured the catalytic ability of “wet” HCPs (i. e., pre‐exposed to humidity) in a pure CO_2_ stream. The HCPs still displayed CO_2_ photoreduction activity (Table S3), albeit decreased, which indicates that CO_2_ could still adsorb onto the materials and react. We note that during the ‘normal’ photoreduction experiments, we did not expose HCPs to water vapor prior to CO_2_ reduction, but rather a stream of CO_2_ containing water vapor.

### CO_2_ photoreduction activity of hypercrosslinked polymers

**Figure 2:**
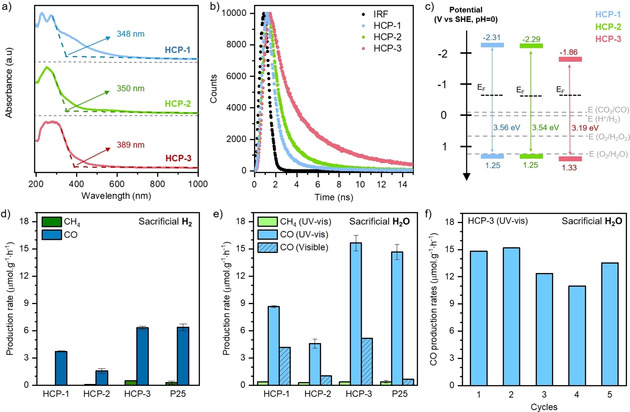
a) UV/Vis absorption coefficient spectra with absorption onsets indicated. b) Photoluminescence decay kinetics probed at 700 nm following excitation at 282 nm, along with the instrument response function (IRF) probed at the excitation wavelength. c) Band structures based on XPS and DRS UV/Vis analyses. d) Photocatalytic production rates using sacrificial H_2_ in UV/Vis light. e) Photocatalytic production rates using sacrificial H_2_O in both UV/Vis and visible light alone. f) HCP‐3 recyclability test over 5 cycles of 3 h irradiation using UV/Vis light and sacrificial H_2_O.

To evaluate the potential of HCPs for CO_2_ photoreduction, we investigated their optoelectronic properties using UV/Vis diffuse reflectance spectroscopy (UV/Vis DRS). The UV/Vis spectra show all HCPs absorb light in both the UV and visible range, with estimated optical gaps of 3.56, 3.54 and 3.19 eV for HCP‐1, HCP‐2 and HCP‐3, respectively (Figure 2a). All three HCPs exhibited photoluminescence above 550 nm (Figure S10). We probed their photoluminescence lifetimes at 700 nm by using time‐correlated single photon counting (TCSPC) upon 282 nm excitation (Figure 2b).

The time at which the photoluminescence signal has decayed to half its initial amplitude (half‐lifetime) was 1.9 ns, 2.2 ns, and 3.2 ns for HCP‐1, HCP‐2, and HCP‐3, respectively, which demonstrates that HCP‐3 has a substantially longer excited state lifetime than HCP‐1 or HCP‐2. We estimated the HCP's valence band (VB) and conduction band (CB) positions by complementing our UV/Vis DRS data with XPS measurements (Figure 2c). First, valence band XPS measurements allowed us to ascertain the distance between the Fermi level (*E*_F_) and the VB onset, also known as VB offset (Figure S11). Placing the VB offset on the absolute energy scale requires knowledge of the position of the Fermi level, which was determined by measuring the secondary electron cut‐off through XPS work function measurements (Figure S12). Finally, we identified the CB position using the UV/Vis spectra absorption onset. For all HCPs, the CB is located above the reduction potential of CO_2_/CO and the VB below the oxidation potential of both H^+^/H_2_ and O_2_/H_2_O. Hence, the band diagrams point towards a sufficient thermodynamic driving force to enable the reduction of CO_2_ to CO using either gaseous H_2_ or H_2_O as a sacrificial agent. The Fermi level of all HCPs lies closer to the CB than the VB, suggesting electrons are the majority charge carriers, which is desirable for CO_2_ photoreduction. Overall, the light absorption properties of HCPs, as well as their CO_2_ adsorption capacities, make them *a priori* attractive candidates for CO_2_ photoreduction.

After establishing their CO_2_ adsorption ability and desirable optoelectronic properties, we tested HCPs for the photocatalytic reduction of gaseous CO_2_. We conducted the tests in a heterogeneous gas/solid photoreactor at ambient temperature, using either H_2_ or H_2_O as a sacrificial agent under UV/Vis or visible irradiation alone (Xe arc lamp, 300 W; Figure S13). No cocatalyst or photosensitizer was required, but some residual iron is present from the HCP synthesis and may play a role in the overall photocatalytic activity (Table S1) as shown with Pd for other organic materials in the context of H_2_ evolution.[^32^, ^33^] However, HCP‐3, the most efficient photocatalyst, has the lowest Fe content (31 ppm). A gas phase reactor was chosen to combine CO_2_ capture and CO_2_ conversion, avoiding limitations owing to poor CO_2_ solubility in water (Figure S1). In each experimental set, we compared HCP performance to that of the benchmark TiO_2_ P25. For all HCPs, after 3 h of irradiation the primary carbonaceous product observed was CO, with a gaseous product selectivity of up to 93 % and 98 % using sacrificial H_2_ or H_2_O, respectively (Figure 2d,e and Tables S3–S5). Trace CH_4_ was also detected, representing the only other gaseous carbonaceous product detected. Gaseous product selectivity is simply the ratio between CO and the sum of all carbonaceous products detected (i. e., CO and CH_4_). Quantum efficiencies were calculated for HCP‐3 in both UV/Vis and visible light alone and compared to a selection of competitive organic photocatalysts for gaseous CO_2_ reduction (Table S5 and S6). We note that this is intended to provide some perspective rather than a direct comparison. To investigate the production of less volatile compounds remaining on the polymer surface, we washed HCP‐3 with water post‐irradiation and analyzed the resulting solution using HPLC. Low concentrations of methanol and formic acid were also detected (Figures S14 and S15), the buildup of which at the surface could lead to decreased catalyst activity with time. Interestingly, when using H_2_O as sacrificial agent, we did not detect O_2_. Instead, we observed H_2_O_2_ after photocatalysis (Figure S16). The formation of H_2_O_2_ may originate from two possible routes. H_2_O might be oxidized first to O_2_ and then reduced to H_2_O_2_ on the surface of the catalyst, resulting in a lack of detectable O_2_. This route has been reported for a different study.^[14]^ Alternatively, H_2_O might be directly oxidized to H_2_O_2_.

Regardless of the sacrificial agent, HCP‐2 exhibited the lowest CO production rate, while HCP‐3 displayed the highest. The photocatalytic performance of HCP‐3 was comparable to that of TiO_2_ P25 under UV/Vis light and was up to 7.5 times better when irradiated with only visible light (Figure 2e and Table S3). In fact, we observed photocatalytic activity for all HCPs under visible light alone, a significant finding for organic materials requiring no doping or cocatalyst. The decrease in activity under visible light compared to UV/Vis is rationalized using their UV/Vis absorption spectra (Figure 2a). As HCP‐1 is the network that shows the highest light absorption in the visible region, its photoactivity is the least affected by the absence of UV light.

Activity in the visible range is promising for the future of HCP photocatalysts in real‐world applications as visible light comprises a large portion of the sun's output reaching the Earth's surface. Targeted network modifications might allow further optimization of this visible light activity. The high photocatalytic activity of HCP‐3 likely arises from the presence of triazine groups. Lee et al. reported stronger electrostatic CO_2_ interactions in triazines when compared to benzene and amino groups, as are present in HCP‐1 and HCP‐2, respectively.^[34]^ Triazine groups may also offer an additional delocalization of the electrons, favoring lower electron‐hole recombination.[^35^, ^36^] Time‐resolved photoluminescence showed that HCP‐3 displayed the slowest electron‐hole recombination rates (Figure 2b), leaving more time for charges to migrate to the surface of the photocatalyst for CO_2_ photoconversion. To gain further insights on the key parameters influencing CO_2_ adsorption and photoconversion, a systematic HCPs screening is required.

**Figure 3:**
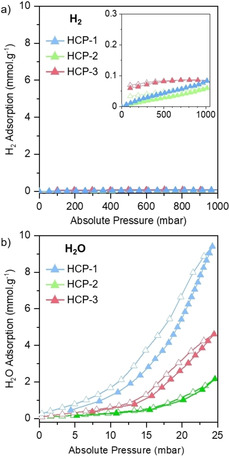
a) H_2_ and b) H_2_O adsorption isotherms at 25 °C. Filled symbols represent adsorption and empty symbols represent desorption. The inset in (a) shows the H_2_ isotherms with a smaller *y* axis for clarity.

Interestingly, CO_2_ photoreduction performance of HCPs improved by up to 2.5 times on replacing H_2_ with sacrificial H_2_O (Figure 2d,e). From a thermodynamic standpoint, such behavior is intriguing as H_2_O oxidation requires a higher driving force than H_2_ oxidation. We hypothesized that the increased photoactivity in the presence of H_2_O is due to HCPs displaying significantly improved adsorption capacities for H_2_O when compared to H_2_. This leads to an increased availability of the sacrificial agent at the photoactive sites on the HCPs’ surface, improving photoreduction rates. To investigate this hypothesis, we collected pure H_2_ and H_2_O adsorption isotherms for all HCPs (Figure 3). From the pure adsorption isotherms, under operating conditions (atmospheric pressure, 25 °C) HCPs adsorbed <0.1 mmol/g of H_2_, in comparison to between 2 and 9.4 mmol/g for H_2_O, an increase of 2 orders of magnitude. HCP‐1 adsorbed the highest amount of H_2_O despite a lack of amino functionality, however, elemental analysis revealed it contained significant residual oxygen from incomplete crosslinking reactions (11.4 wt% compared to 4.1 and 3.6 wt% for HCP‐2 and HCP‐3, respectively), likely increasing its H_2_O adsorption ability. Adding to this effect, the high porosity and particularly microporosity will also contribute to the water uptake of the HCPs, thereby explaining the trend in water sorption. A higher concentration of sacrificial agent at the HCP's surface should favor high CO_2_ photoreduction rates, explaining the increase of photoactivity when using H_2_O as reducing agent.

To further investigate the importance of H_2_O adsorption on the photoactivity, we decreased the humidity inside the photoreactor by decreasing the temperature of the H_2_O vapor saturator to around 1 °C. At 1 °C, the partial pressure of H_2_O at saturation is reduced (6.6 mbar), thereby dramatically reducing the concentration of H_2_O introduced into the system. After sealing the reactor, we conducted CO_2_ photoreduction measurements at 25 °C, under atmospheric pressure. The decreased humidity of the stream resulted in a 38 % decrease in activity for HCP‐3 (Figure S17). Indeed, H_2_O uptake in this network is reduced to around 0.4 mmol/g at 6.6 mbar, as estimated from the pure water sorption isotherm (Figure 3b). This further corroborates the importance of the adsorption of the sacrificial agents to the photocatalyst surface. TiO_2_ also showed improvements when employing sacrificial H_2_O in place of H_2_. Sorescu et al. outlined how co‐adsorbed water on TiO_2_ can positively affect the adsorption capacities of CO_2_ through the formation of hydrogen bonds.^[37]^ Other materials such as ZrO and coal also exhibit increased CO_2_ adsorption and/or activation in the presence of co‐adsorbed H_2_O.[^38^, ^39^, ^40^] In addition to ensuring the presence of H_2_O molecules close to active sites, we speculate that co‐adsorbed water may also favor CO_2_ activation and/or the formation of bicarbonate species and facilitate its photoreduction, as suggested elsewhere for other photocatalysts.[^41^, ^42^] Nonetheless, further mechanistic studies are required to understand the influence of H_2_O on CO_2_ adsorption and the structure‐property relationship for these materials.

We examined further the photocatalytic properties under UV/Vis irradiation of HCP‐3, the most active photocatalyst of this study. Kinetic studies point to a relatively linear production of CO up to 3 h of irradiation, which decreases slightly after 6 h (Figure S18). Recyclability tests were also performed. After 5 cycles the photoactivity decreased by 9 % (Figure 2f), which may be due to the formation of surface products (e. g., methanol and formic acid) or mild degradation of the polymer.

To assess the possible degradation, we collected XPS spectra of all polymers before and after irradiation. Deconvolution of C1s, N1s and O1s spectra for all HCPs showed that the amount of sp^3^ aliphatic crosslinker and the amount of partial crosslinked C−O slightly decreased after irradiation (Figures S19–S21). Elemental analysis showed minimal change in bulk polymer composition after the irradiation process (Table S1). We also collected ssNMR spectra for all networks after the photoreduction process (Figure S3), which also showed no major structural change to bulk composition. We then probed the structural and chemical stability of HCP‐3 further by collecting N_2_, CO_2_, and H_2_O sorption isotherms and FTIR spectra, all of which showed no significant alteration, with the exception of a change in the shape of the N_2_ isotherm (Figures S22 and S23 and Table S2). The slight degradation of the polymer seen in XPS measurements post‐irradiation may arise from the formation of H_2_O_2_ which decomposes into hydroxyl radicals under UV/Vis irradiation and leads to structural changes.

To verify the evolution of CO from CO_2_ conversion over HCPs photocatalysts, we conducted a series of control experiments, in an inert atmosphere (N_2_/H_2_ or N_2_/H_2_O), without catalyst, without light, and with isotopic labeling of ^13^CO_2_ (details of control experiments are given in Table S3). In the absence of CO_2_, the activity decreased by 77 % to 88 % depending on the atmosphere (N_2_/H_2_O vs N_2_/H_2_). We attribute the trace CO detected under inert atmosphere to the degradation of some carbon sp^3^ aliphatic crosslinker and residual oxygen‐containing functional groups of the HCP crosslinkers. To verify the photocatalytic production of CO from CO_2_, we conducted isotopic labeled ^13^CO_2_ tests using H_2_O as a sacrificial agent under UV/Visible light irradiation. We observe a ^13^CO peak (*m*/*z*=29) after light irradiation, confirming the ability of HCPs to photoconvert CO_2_ to CO (Figure S24). No ^13^CH_4_ was observed, which is likely due to the good selectivity of the HCP toward CO (Figure 2d,e).

## Conclusion

We have reported herein hypercrosslinked polymers (HCPs) for the first time as a photocatalytic platform for CO_2_ photoreduction under both UV/Vis and visible light irradiation. HCPs show promising photocatalytic activity using only sacrificial H_2_O, without the requirement for any cocatalyst or photosensitizer, significantly outperforming the benchmark material TiO_2_ P25 under visible light illumination. This was rationalized by new insights into the concentration of sacrificial agents at the surface of HCPs through selective adsorption, as networks showed significantly higher H_2_O adsorption capacity in comparison to negligible H_2_ adsorption. The performance disparity between these reducing agents outlines a key consideration when producing photocatalysts for yield efficient solar‐energy conversion. Their lack of requirement for precious‐metal catalysts, as well as their simple engineering, good general stability, and low cost, make HCPs an exciting and promising platform for the design of organic photocatalysts.

## Experimental Section

### Materials

TiO_2_ P25 (>99.5 %, 21 nm primary particle size), benzene, dimethoxymethane, 2,4‐diamino‐6‐phenyl‐1,3,5‐triazine, aniline, 1,2‐dichloroethane, N,N‐dimethylformamide and iron(III) chloride were all purchased from Sigma‐Aldrich. 4,4′‐bis(methoxymethyl)biphenyl was purchased from Tokyo Chemical Industry (TCI) UK and methanol (reagent grade) was purchased from VWR. All reagents were used as received. All gases were purchased from BOC.

### Hypercrosslinked Polymer Synthesis

HCP‐1: Anhydrous 1,2‐dichloroethane (20 mL) was added to benzene (0.78 g, 10 mmol) and dimethoxymethane (2.28 g, 30 mmol) under N_2_, before the solution was purged with N_2_ for at least a further 30 min. After purging, iron(III) chloride (4.87 g, 30 mmol) was quickly added to the solution and the mixture was heated to 80 °C for a further 24 h, during which the reaction was kept under an inert atmosphere. The resulting solid was allowed to cool before it was filtrated using a Büchner funnel and washed with methanol until the filtrate was almost colorless. The polymer was then further washed by Soxhlet extraction in methanol for 24 h. Finally, the polymer was dried in a vacuum oven overnight at 70 °C.

HCP‐2: The overall procedure remained the same as HCP‐1 with 1,2‐dichloroethane (20 mL) added to aniline (0.28 g, 3 mmol) and 4,4′‐bis(methoxymethyl)biphenyl (1.45 g, 6 mmol) before iron(III) chloride (0.98 g, 6 mmol) was added.

HCP‐3: Again, the overall procedure remained the same as HCP‐1 with 1,2‐dichloroethane (20 mL) added to 2,4‐diamino‐6‐phenyl‐1,3,5‐triazine (0.37 g, 2 mmol) and 4,4′‐bis(methoxymethyl)biphenyl (0.97 g, 4 mmol) before iron(III) chloride (0.65 g, 4 mmol) was added. In addition to methanol, HCP‐3 was also washed in chloroform by Soxhlet extraction, to ensure the removal of unreacted 2,4‐diamino‐6‐phenyl‐1,3,5‐triazine. Yields for HCP‐1, HCP‐2 and HCP‐3 were 84 %, 66 % and 91 %, respectively, based on hypothetical 100 % polycondensation.

All samples were fully characterized and details about the characterization techniques can be found in the Supporting Information.

### Photocatalytic measurements

A gas/solid photoreactor was assembled to conduct CO_2_ photocatalytic measurements (Figure S1). Tests were conducted at ambient temperature. The photocatalysts were deposited on a stainless‐steel plate with a fixed area of 9.6 cm^2^. To do so, 10–15 mg of the ground photocatalyst was dispersed in DI water (1.2 mL), sonicated for 30 sec and drop cast onto the sample holder. Research grade (99.999 %) CO_2_ and H_2_ (99.9995 %, Peak Scientific PH200 hydrogen generator) were flowed at controlled rates using mass flow controllers (Omega Engineering, 0–50 mL min^−1^). For experiments using H_2_ as sacrificial agent, the photoreactor (35 cm^3^) was vacuumed and replenished with a gas mixture of CO_2_ and H_2_ (1.5 vol/vol ratio) six times. The same gas mixture of CO_2_ and H_2_ was subsequently passed over the catalyst bed in the photoreactor for 15 residence times before it was sealed at 1.25 bar and irradiated for 3 h. For experiments using water as sacrificial agent, H_2_O vapor was generated from a saturator at room temperature (25 °C), unless stated otherwise, providing a gas mixture of CO_2_ and H_2_O (1.4 wt% H_2_O). The photoreactor was vacuumed and replenished with a gas mixture of CO_2_ and H_2_O at least six times prior to measurement. A xenon arc lamp (300 W, *λ*>325 nm, LOT Quantum Design) equipped with a water filter was used as the irradiation source. The distance from the lamp to the sample was 9.5 cm with a broadband irradiance at the sample surface of 1830 W m^−2^. For visible light measurements, a long‐pass UV filter (*λ*<400 nm; LOT Quantum Design) was used.

Evolved gases were analyzed by a gas chromatograph (GC) with gas sampling valves connected directly to the photoreactor. The GC (Agilent Technologies, 7890B) was equipped with HayeSep (Agilent J&W 6 foot, 1/8 inch, 2 mm, HayeSep Q Column 80/100 SST) and molecular sieve (Agilent J&W 6 foot, 1/8 inch, 2 mm, MolSieve 5 A, 60/80, preconditioned) packed columns in series, a thermal conductivity detector (TCD) and a flame ionization detector (FID). For recyclability tests, the aforementioned process was repeated after each 3 h irradiation cycle without opening the photoreactor. In addition, isotopic tracing experiments were performed with ^13^CO_2_ (BOC, >98 % atom ^13^CO_2_ compared to ^12^CO_2_, >99 %). The reactor was flushed with He for 1 h before injecting 15 mL of ^13^CO_2_. After irradiation, the evolved gases were analyzed by a mass spectrometer (Shimadzu MS) equipped with a Q‐bond and a MolSieve column with gas sampling valves connected directly to the photoreactor. The photocatalytic CO_2_ reduction tests were repeated 3 times for each material under the same reaction conditions.

## Conflict of interest

The authors declare no conflict of interest.

## Supporting information

As a service to our authors and readers, this journal provides supporting information supplied by the authors. Such materials are peer reviewed and may be re‐organized for online delivery, but are not copy‐edited or typeset. Technical support issues arising from supporting information (other than missing files) should be addressed to the authors.

Supplementary
