# Supplementary material for: Hypercrosslinked Polymers as a Photocatalytic Platform for Visible‐Light‐Driven CO2 Photoreduction Using H2O
Source: ChemSusChem. 2021 Jan 22;14(7):1720–7. doi: 10.1002/cssc.202002824 (PMC8048809; doi:10.1002/cssc.202002824)
Supplement: Supplementary file 1 — Supplementary [file CSSC-14-1720-s001.pdf]

ChemSusChem

Supporting Information

Hypercrosslinked Polymers as a Photocatalytic Platform for Visible-Light-Driven CO_2 Photoreduction Using H_2O

Giulia E. M. Schukraft, Robert T. Woodward,* Santosh Kumar, Michael Sachs, Salvador Eslava, and Camille Petit*© 2021 The Authors. ChemSusChem published by Wiley-VCH GmbH. This is an open access article under the terms of the Creative Commons Attribution License, which permits use, distribution and reproduction in any medium, provided the original work is properly cited.

Table of Contents

1. **Characterisation** S1
2. **Supplementary Figures and Tables** S6
3. **References** S39

1. Characterisation

Chemical and structural properties

Solid-state NMR was carried out on a Bruker Avance NEO 500 wide bore system (Bruker BioSpin, Rheinstetten, Germany) using a 4 mm triple resonance magic angle spinning probe. Around 15 to 25 mg of material was packed into a 4 mm zirconia CRAMPS rotor. The resonance frequency for ^{13}C NMR was 125.78 MHz, the MAS rotor spinning was set to 14 kHz. Cross polarization was achieved by a ramped contact pulse with a contact time of 3 ms. During acquisition ^{1}H was high power decoupled using SPINAL with 64 phase permutations. The ^{1}H $\pi/2$ pulse was 2.5 µs, the relaxation delay was set to 4 s, and with roughly 2000 scans a sufficient signal to noise could be achieved. The chemical shifts for ^{13}C are reported in ppm and are referenced external to adamantane by setting the low field signal to 38.48 ppm.

Fourier-transform infrared (FTIR) spectroscopy was performed in the range of 500 – 4000 cm^{-1} on finely ground samples using a PerkinElmer Spectrum 100 FT-IR spectrometer equipped with an attenuated total reflectance (ATR) accessory.

Thermal analyses were performed using a Netzsch TG209 F1 Libra thermogravimetric analyser. At least 10 mg of sample was heated from room temperature to 900 °C at a rate of 10 °C min^{-1} under either air or N_2 gas flow (flow rate 100 mL min^{-1}). An initial isothermal step of 1 h was included at 120 °C to ensure removal of adsorbates before heating continued.

X-ray photoelectron spectroscopy (XPS) measurements were carried out on a Thermo Scientific K-Alpha+ X-ray photoelectron spectrometer equipped with a MXR3 Al Kα monochromated X-ray source (hν = 1486.6 eV). Samples were ground and mounted on the XPS holder using a conductive carbon tape. The X-ray gun power was set to 72 W (6 mA and

12 kV). Survey scans were acquired using 200 eV pass energy, 0.5 eV step size, and 100 ms (50 ms x 2 scans) dwell times. Data analysis was performed using the Thermo Avantage data analysis program.

Nitrogen isotherms were measured using a porosity analyser (Micromeritics 3Flex) at -196 ^{o}C. Prior to measurement, all samples were degassed overnight at 393 K at around 0.2 mbar pressure. Samples underwent a further degas step at 393 K *in-situ* on the porosity analyser for 4 h, this time at around 0.003 mbar. Surface areas were calculated using the Brunauer−Emmett−Teller (BET) method.[1] The total volume of pores (V_{TOT}) was calculated from the volume of N_2 adsorbed at $P/P_0 = 0.97$ and micropore volume (V_{MICRO}) was determined using the Dubinin−Astakhov method.[2] The pore size distribution was derived from the adsorption isotherms by using an built-in software from Micromeritics and selecting the DFT model for carbon slit shape pores (N_2@77 on Carbon Slit Pores by NLDFT).

Powder X-ray diffraction (PXRD) measurements were recorded at room temperature on a BRUKER 2D PHASE diffractometer operating at 30 kV and 10 mA with monochromatised Cu Kα radiation (λ = 0.15418 nm).

The morphology of the samples was imaged using a scanning electron microscope (SEM, Leo Gemini 1525, Zeiss) in secondary electron mode (InLens detector) at 5 kV. The samples were ground, deposited on carbon tape, and coated with 20 nm of chromium to reduce charging.

For determination of iron content in HCP-1,-2 and -3, 30 mg of sample were added to 8 mL of sulfuric acid. The resulting mixture was heated on a hot plate until the boiling point of sulfuric acid boiling was reached. Then, 4 mL of $HClO_4$ was added dropwise to oxidise the sample to complete digestion. Digested samples were analysed using a Varian 720 ES with simultaneous ICP-OES.

Elemental analysis was performed using a Eurovector EA 3000 CHNS-O Elemental Analyser. Between 0.75 and 3.0 mg of each sample was weighed into tin vials (4×6 mm) for each individual run and each sample was ran at least in duplicate. Sample weighing is done using a micro balance (Sartorius, ME 5 OCE) to ensure accuracy. The operating temperatures for the combustion and reduction were 1000 °C (1480 °C for O analysis) and 750 °C, respectively, with high purity helium (99.999+) used as a carrier gas.

Optoelectronic properties

Valence band X-ray photoelectron spectroscopy (XPS) and work function measurements were carried out on a Thermo Scientific K-Alpha$^+$ X-ray photoelectron spectrometer equipped with a MXR3 Al Kα monochromated X-ray source (hν = 1486.6 eV). Samples were ground and mounted on the XPS holder using a conductive carbon tape. The X-ray gun power was set to 72 W (6 mA and 12 kV). Valence band spectra were obtained using 15 eV pass energy and 0.05 eV step size. Data analysis was performed using the software Thermo Avantage. The work functions of the polymers were determined by measuring the secondary electron cut-off in the low kinetic energy region. The sample holder contained a clean gold standard sample, which was used as a reference material to ensure correct calibration. A sample bias of -29.47 V was applied to the samples using an ion gun and the cut-off spectra were obtained using a pass energy of 10 eV. To account for potential variations across the surface of the material, the work function was measured at three different locations and the average was taken. A total standard deviation of ± 0.04 eV is associated to the band edge positions. To convert the valence band position and the work function to the absolute energy scale vs. vacuum with the redox potential scale vs. SHE, a factor of 4.44 was required, as 4.44 eV on the former corresponds to 0.00 V on the latter, at 25 °C.

Diffuse reflectance ultraviolet-visible (DR-UV-Vis) spectra were obtained using a Perkin-Elmer Spectrum 100 Spectrometer equipped with an integrating sphere. Spectral band width was set to 2 nm, with Spectralon as a standard.

Time-correlated single photon counting (TCSPC) experiments were carried out using a commercial TCSPC setup (Horiba DeltaFlex) equipped with a pulsed LED excitation source (Horiba NanoLED series) and a fast rise-time photomultiplier detector (Horiba PPD-650 and PPD-900). The instrument response function (IRF) was measured at the wavelength of the excitation source (282 nm). During all other measurements, a suitable long pass filter was inserted between sample and detector to block off scattered excitation light.

Gas and water sorption

Water vapor, CO_2 and H_2 adsorption isotherms were collected at 25 °C using a Micromeritics 3Flex instrument, equipped with a liquid container in the case of H_2O. HCPs were degassed overnight at 120 ^{o}C at around 0.2 mbar pressure and again *in-situ* on the porosity analyser (Micromeritics 3Flex) for 4 h down to around 0.003 mbar. Research grade (99.999%) CO_2 and H_2 (99.9995%, Peak Scientific PH200 hydrogen generator) were used for CO_2 and H_2 isotherms. For H_2O isotherms, miliQ water with a resistance > 18.2 micro-ohms was purified by 4 freeze pump thraw cycles. Water isotherms were collected up to a relative pressure of 0.8 to avoid condensation. For "wet" CO_2 uptake, i.e. investigating CO_2 uptake on HCPs pre-exposed to H_2O, HCPs were exposed to humid air (>99 % humidity) by placing HCPs in a sealed vessel containing liquid water and a hygrometer for at least 48 hours at room temperature. HCPs were not in contact with the liquid water during this process. After removal, CO_2 adsorption isotherms were performed at 25 °C up to 1 bar, skipping all prior degas steps. The first pressure CO_2 adsorption point was collected at around 10 mbar (~2 orders of

magnitude higher than a standard ‘dry’ measurement) to minimise water desorption. Resulting isotherms for wet polymers gave negative adsorption values at low absolute pressures due to some water desorption in the initial stages of measurement. Therefore, to allow comparison to dry samples, a factor was applied to the isotherm, raising the lowest absolute pressure measurement to 0 mmol/g adsorbed CO_2. It is worth noting that some subsequent uptake may be due to re-adsorption of desorbed water.

Photocatalytic properties

H_2O_2 was detected using a colorimetric peroxide test stick from sigma Aldrich. 20 mg of HCP-1 was disperse into 12 mL of H_2O. The photoreactor was vacuum 3 times and backfilled with Research grade (99.999%) CO_2 for 1h to reach the adsorption/desorption equilibrium of CO_2 on the catalyst surface. The photoreactor was then irradiated for 3 h under UV-vis light. After irradiation, HCP-3 was filtered and removed from the solution. A peroxide test stick was then dipped 1 seconds into the aqueous solution. In presence of H_2O_2 a colour change from white to blue can be observed (see Figures S16).

Formic acid and methanol were quantified using HPLC. After 3 h of UV-vis irradiation, the sample was dispersed in 2 mL of water for 10 min. After immersion, HCP-3 was filtered and removed from the aqueous solution. 20 µl of the filtrate was then analysed by HPLC (instrument: Shimadzu Prominence 20A, column: SUPELCOGEL C-610H, 30 cm x 7.8 cm with Supelguard 610H, mobile phase: 0.1% (v/v) phosphoric acid, flow rate: 0.6 mL/min, temp: 30 °C, sample volume: 20 µl, run time: 23 min).

2. Supplementary Figures and Tables

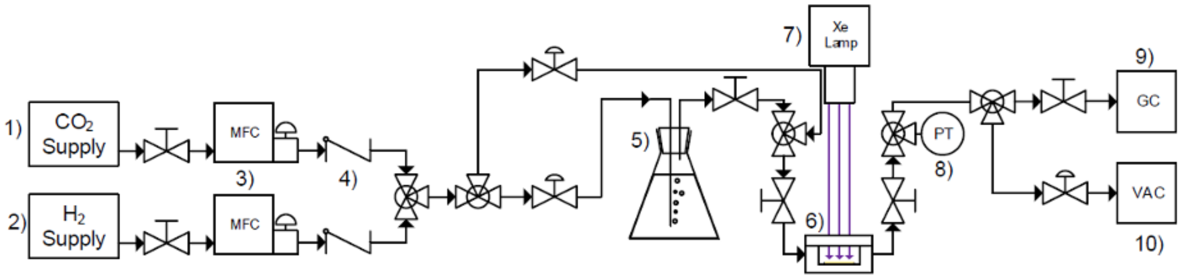

Figure S1. Photocatalytic gas-solid reactor setup used to evaluate photocatalytic CO_2 reduction: 1) CO_2 cylinder, 2) H_2 generator, 3) mass flow controllers, 4) non-return valves, 5) H_2O saturator, 6) photoreactor, 7) Xe arc lamp, 8) pressure transducer, 9) gas chromatograph, 10) vacuum pump. Adapted from ref. [3]

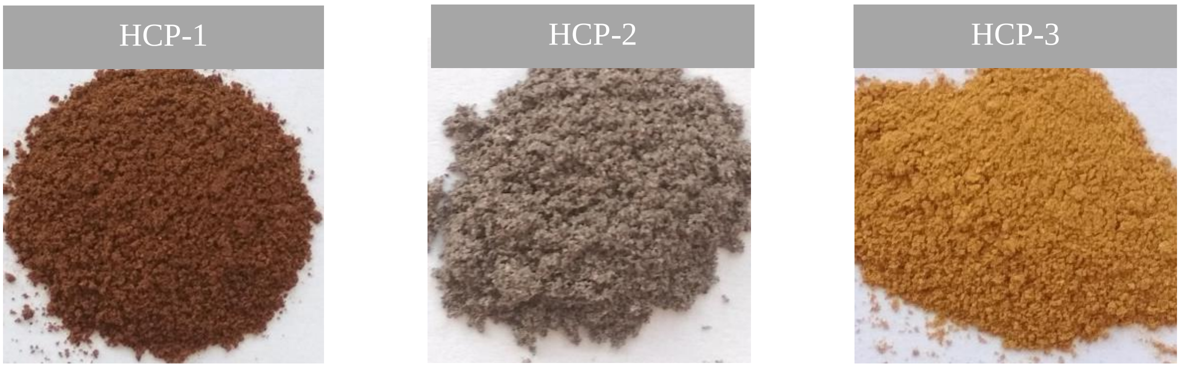

Figure S2. Photographic images of: HCP-1, HCP-2 and HCP-3 (from left to right, respectively).

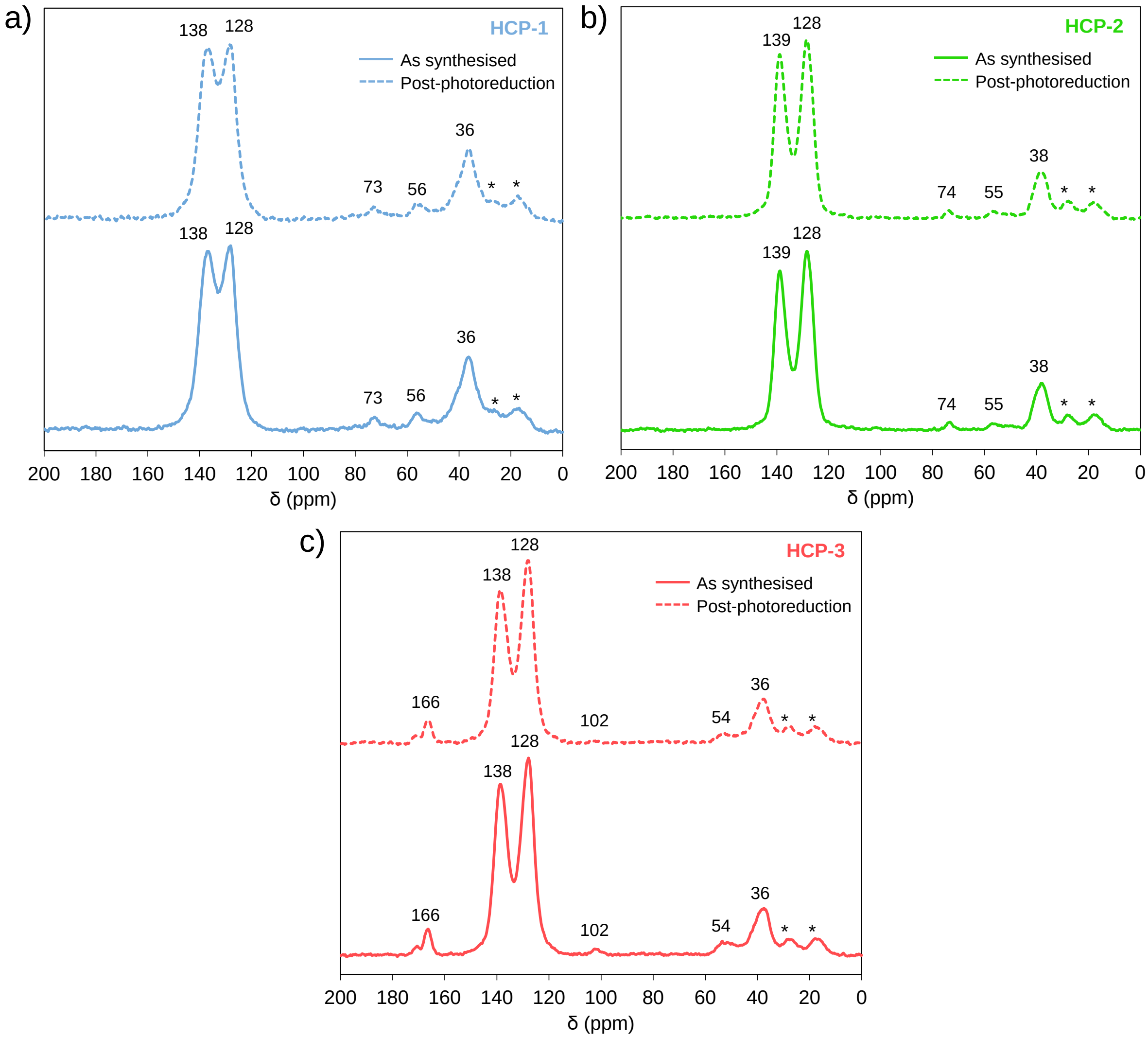

Figure S3. Solid-state NMR spectra of networks as synthesised and post CO_2 photoreduction, a) HCP-1, b) HCP-2, and c) HCP-3. Spinning sidebands are labelled with an asterisks.

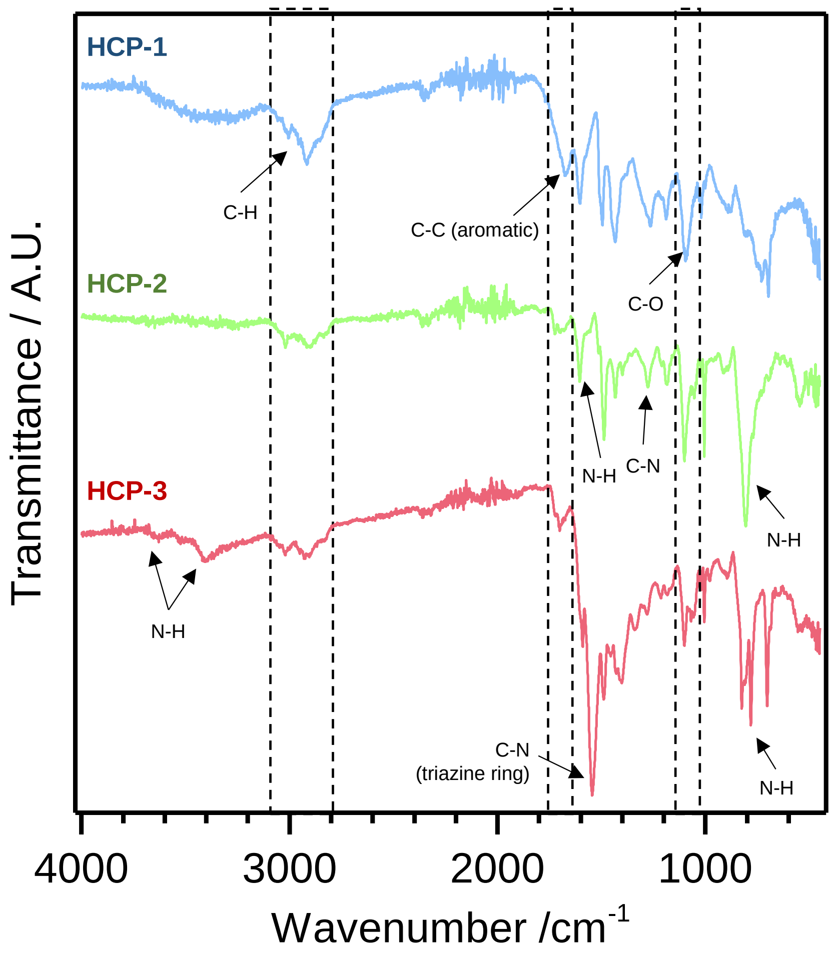

Figure S4. FTIR spectra of HCP-1 (blue), HCP-2 (green) and HCP-3 (red). Signals present in all spectra are highlighted by dashed lines.

Table S1. Atomic composition of HCP-1, HCP-2, and HCP-3 before and after irradiation as well as residual Fe content for all networks.

	O [post-irradiation] (wt.%)[a]	N [post-irradiation] (wt.%)[a]	C [post-irradiation] (wt.%)[a]	Fe (ppm)[b]
HCP-1	11.38 [10.29]	- [-]	76.09 [78.81]	14500
HCP-2	4.12 [4.83]	7.05 [7.06]	81.47 [81.03]	487
HCP-3	3.55 [3.89]	1.34 [1.47]	88.82 [88.27]	31

[a]Determined using CHNS-O analysis. [b]Determined using ICP-MS. Figures in square brackets refer to data measured for polymers post-irradiation.

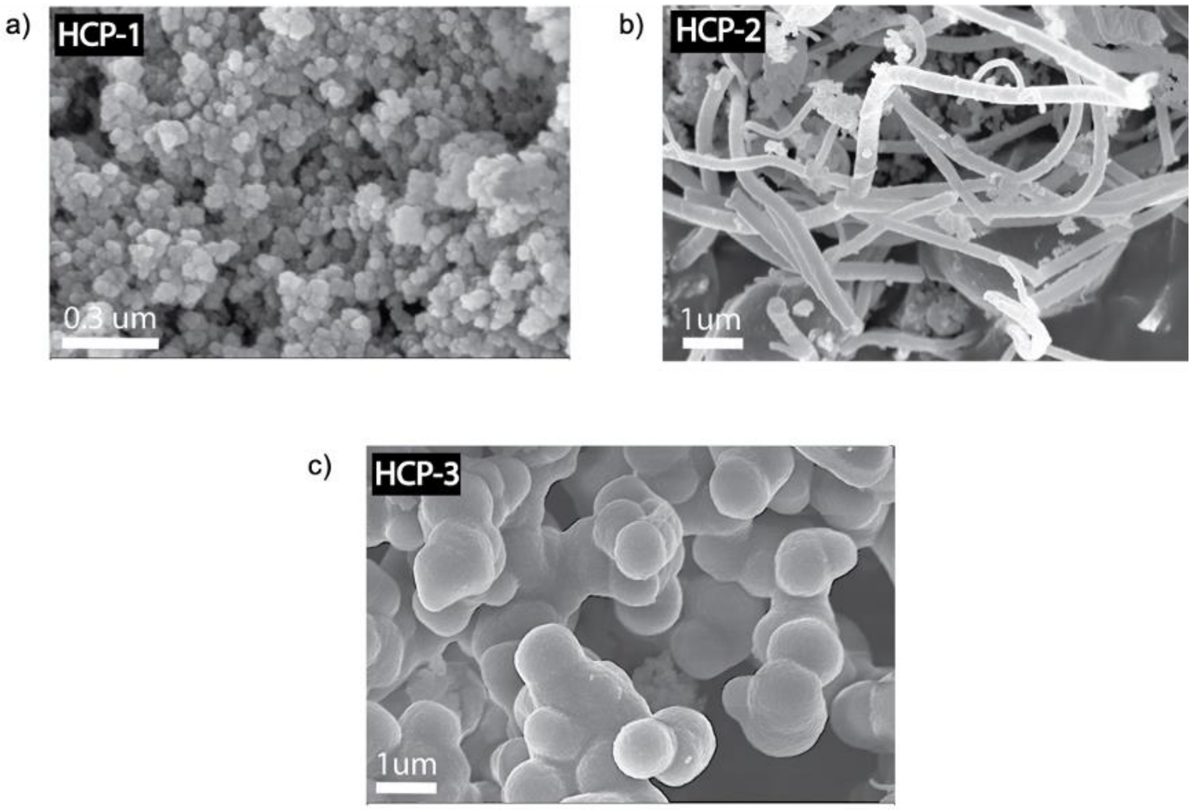

Figure S5. SEM images of a) HCP-1, b) HCP-2, and c) HCP-3.

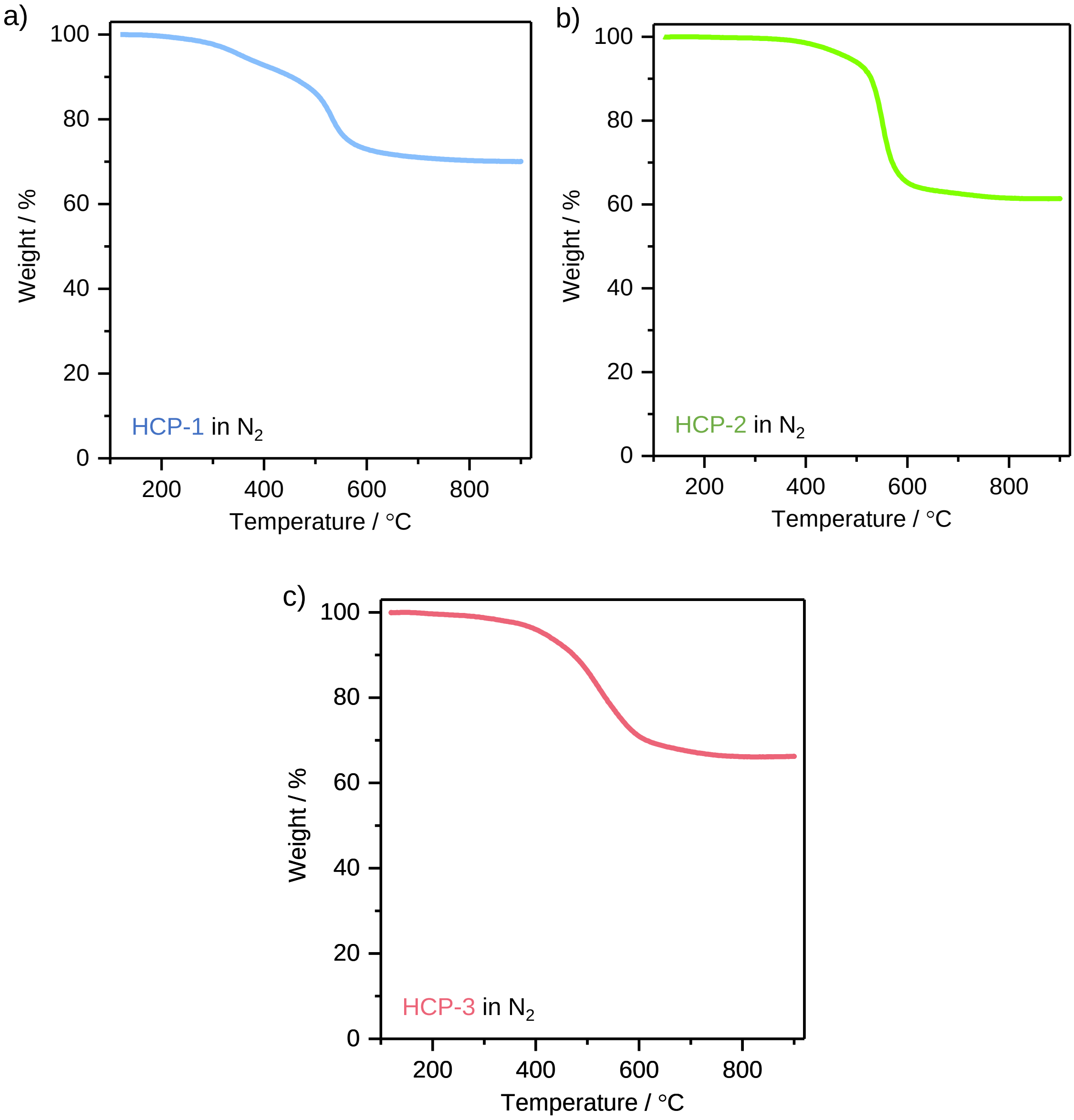

Figure S6. TGA thermograms up to 900 °C in a N_2 atmosphere, heated at a ramp rate of 10 °C/min with a N_2 flow rate of 100 mL/min. a) HCP-1, b) HCP-2, and c) HCP-3.

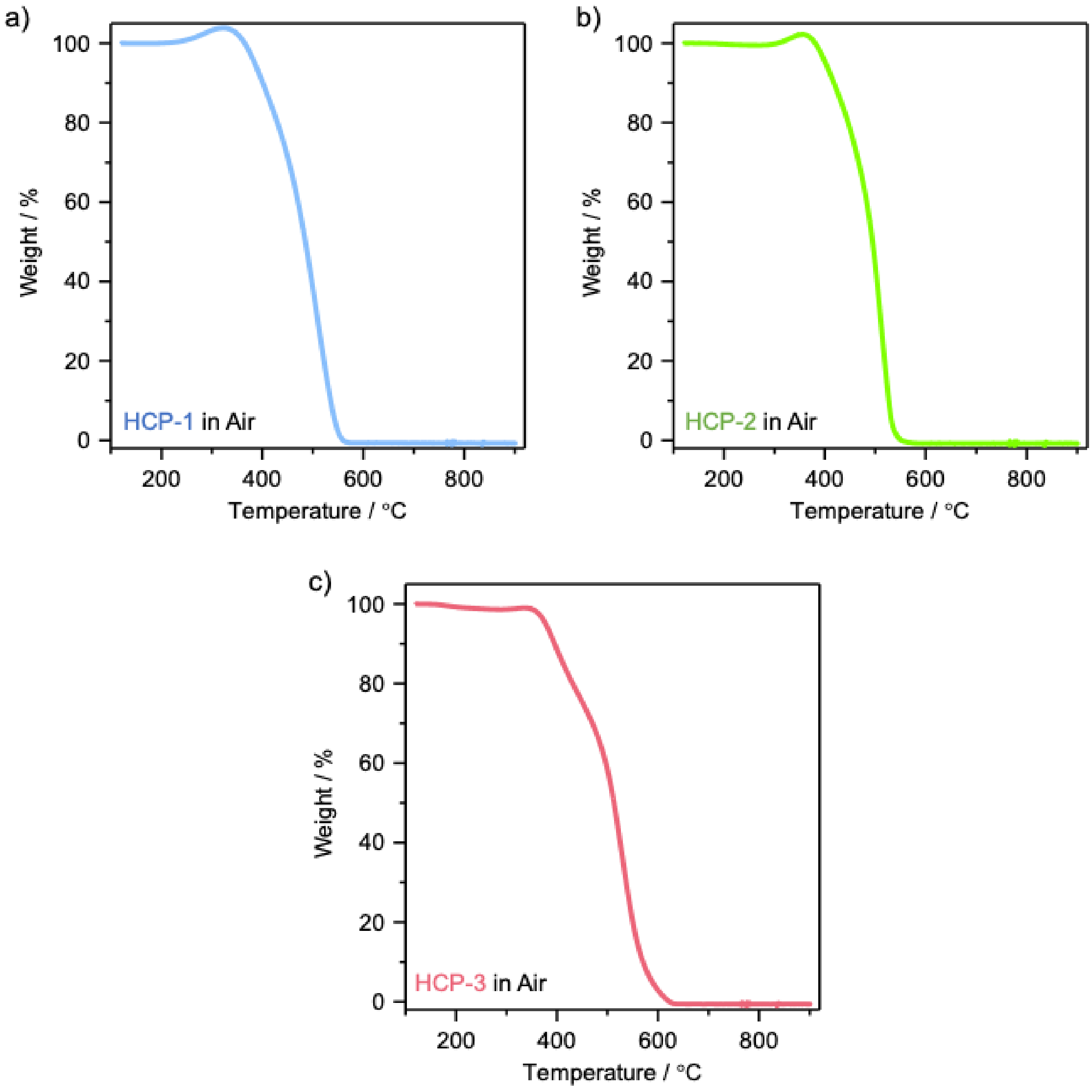

Figure S7. TGA thermograms up to 900 °C in air, heated at a ramp rate of 10 °C/min with an air flow rate of 100 mL/min. a) HCP-1, b) HCP-2, and c) HCP-3.

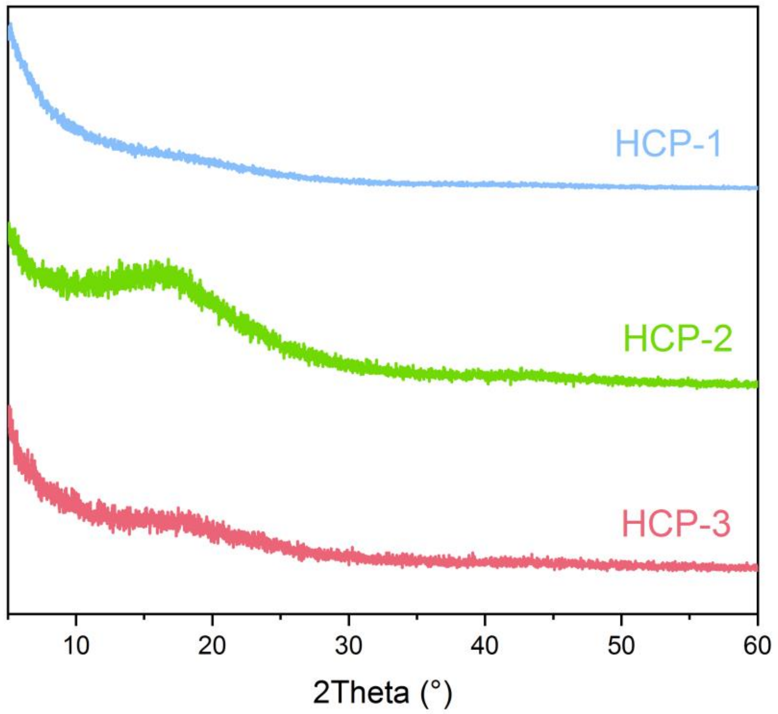

Figure S8. PXRD patterns of a) HCP-1, b) HCP-2, and c) HCP-3.

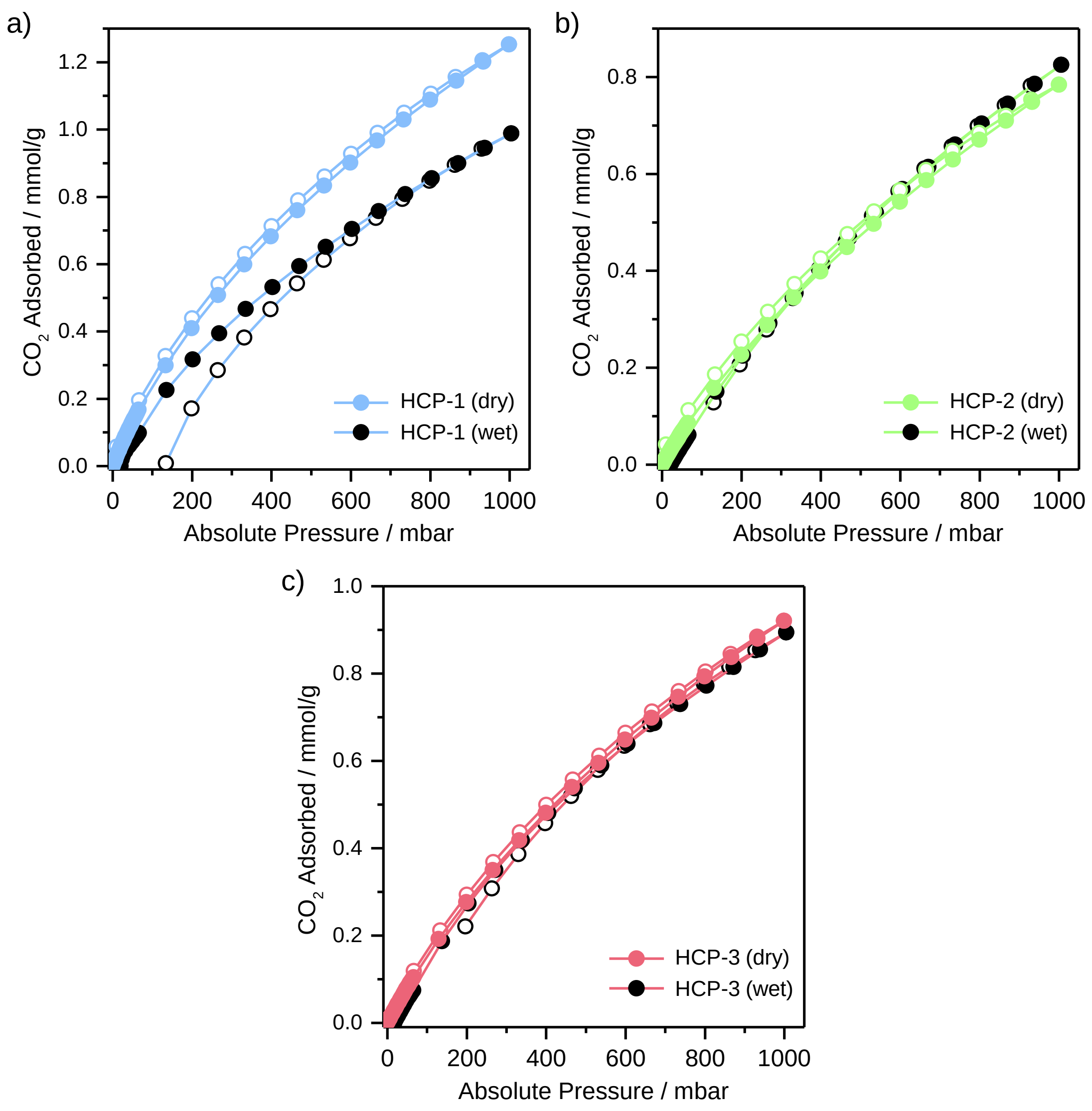

Figure S9. CO_2 adsorption isotherms at 298 K of both dry and wet (i.e. exposed to humid air for at least 48 h before measurement, 99% humidity) networks. a) HCP-1, b) HCP-2, and c) HCP-3. Filled symbols represent adsorption, empty symbols represent desorption.

Table S2. Textural parameters derived from N_2 sorption isotherms at –196 ^oC and CO_2 adsorption capacities at 25 ^oC.

Sample	V_{MICRO} (cm^3/g)[a]	V_{TOT} (cm^3/g)[a]	SA_{BET} (m^2/g)[a]	CO_2 ad. (mmol/g)	
				Dry[b]	Wet[c]
HCP-1	0.46	1.07	950	1.25	0.99
HCP-2	0.13	0.21	310	0.78	0.82
HCP-3	0.16	0.25	360	0.92	0.89
HCP-3 after irradiation	0.11	-	300	0.77	-

[a]Derived from N_2 sorption isotherms at – 196 ^oC .
[b]Capacity of degassed HCP at 25 ^oC and 1 bar
[c]Capacity of non-degassed HCP at 25 ^oC and 1 bar after 48 h exposure to humid atmosphere

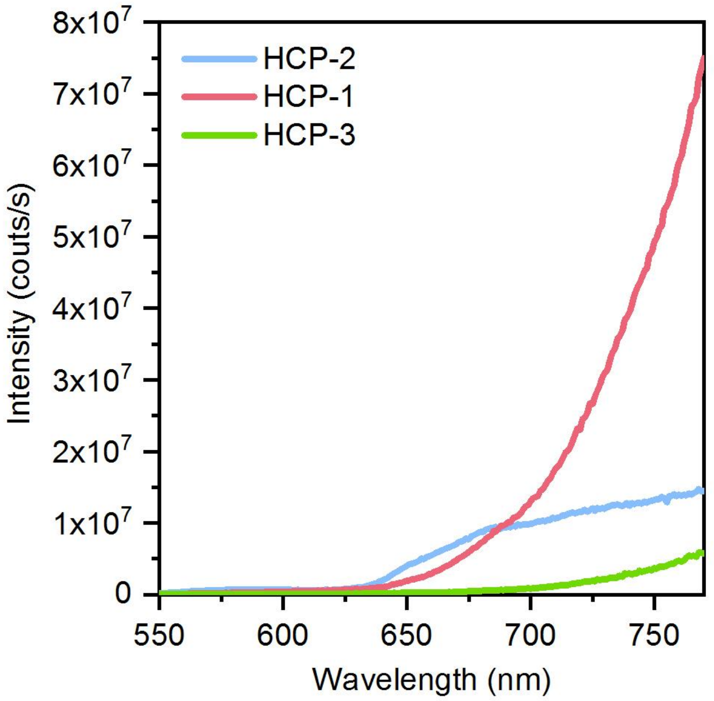

Figure S10. Photoluminescence emission spectra

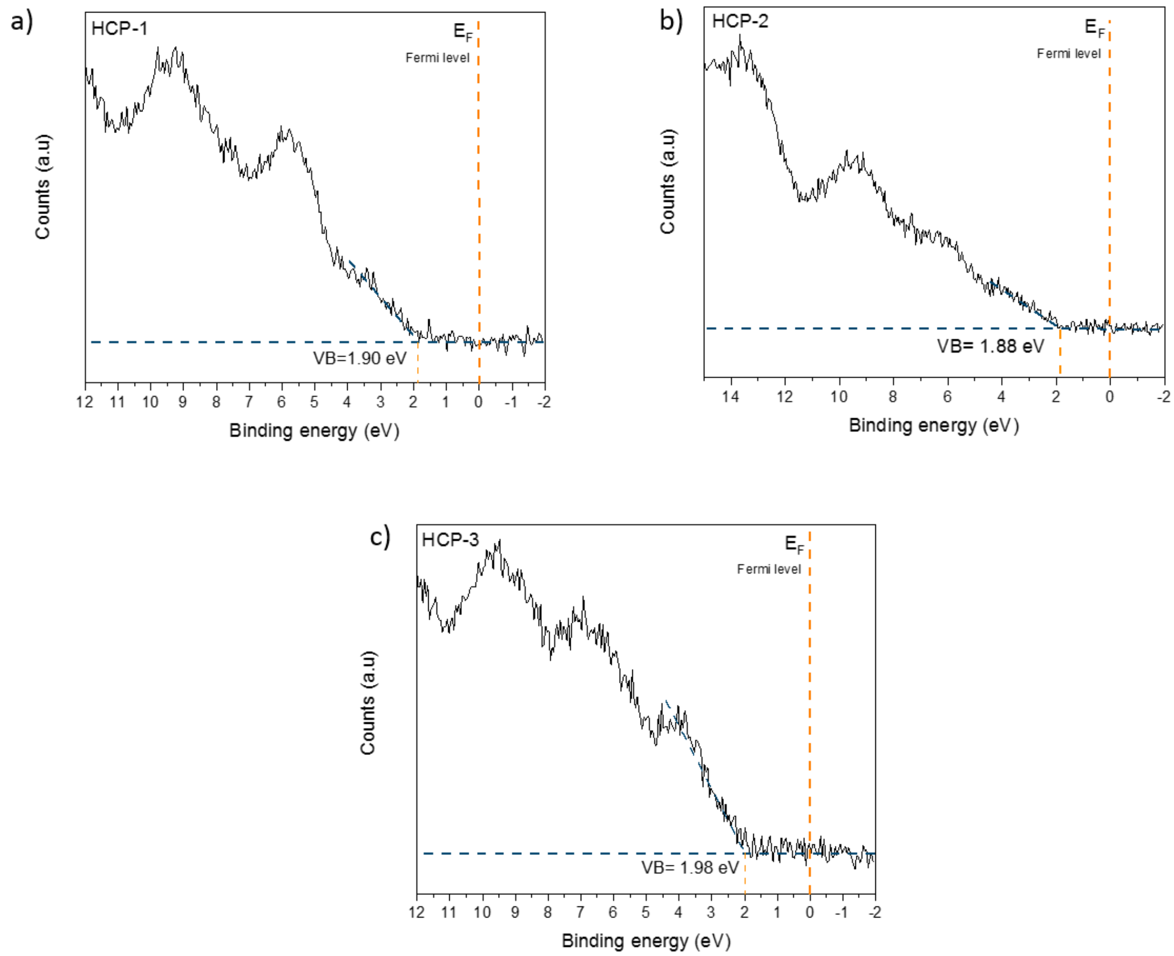

Figure S11. Valence band XPS spectra of a) HCP-1, b) HCP-2, and HCP-3. To account for potential variation on the surface valence band measurements were collected on an area of 4 different points.

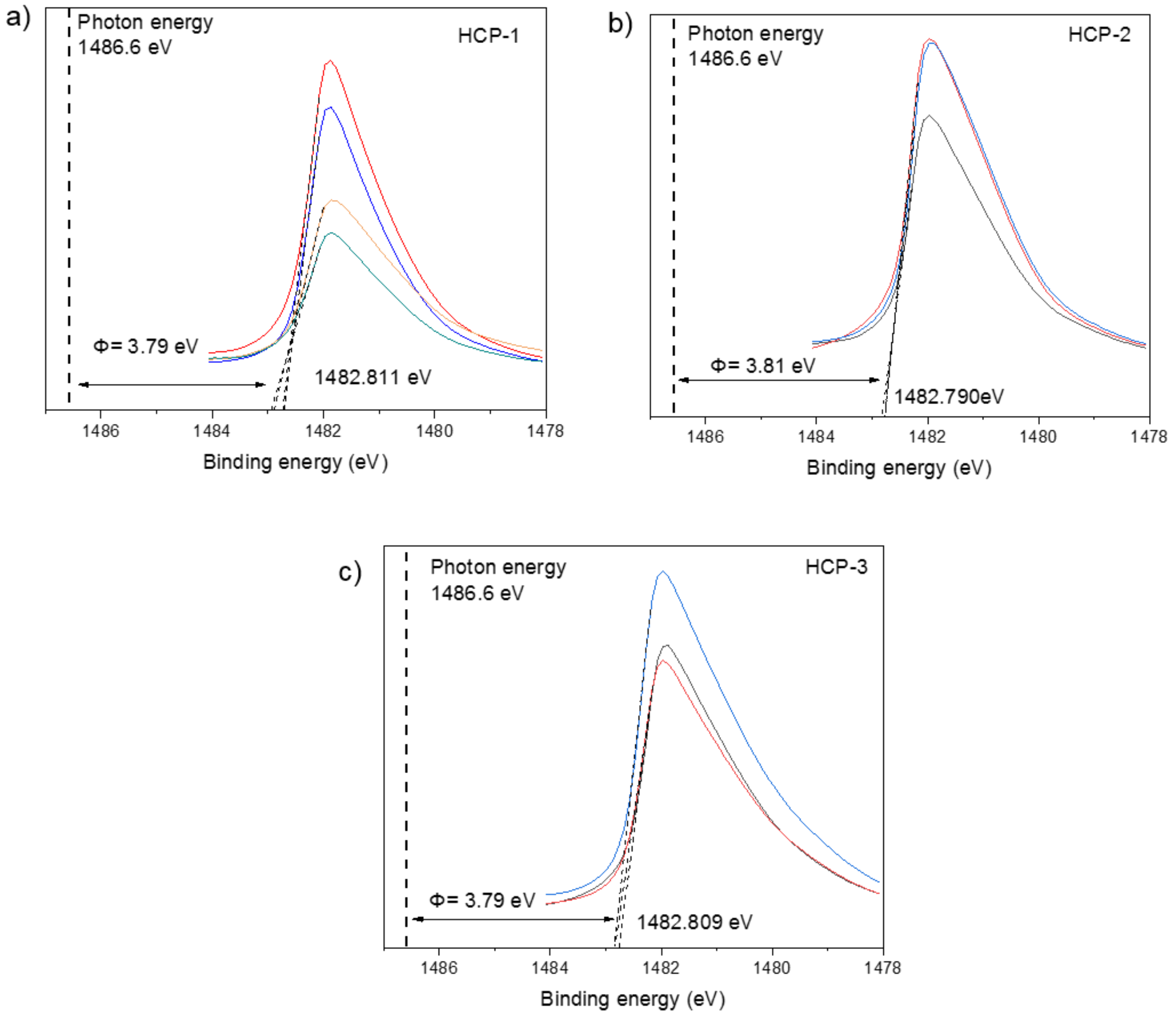

Figure S12. Work function measurements at different locations on a) HCP-1, b) HCP-2, and c) HCP-3 to account for potential variations across the surface.

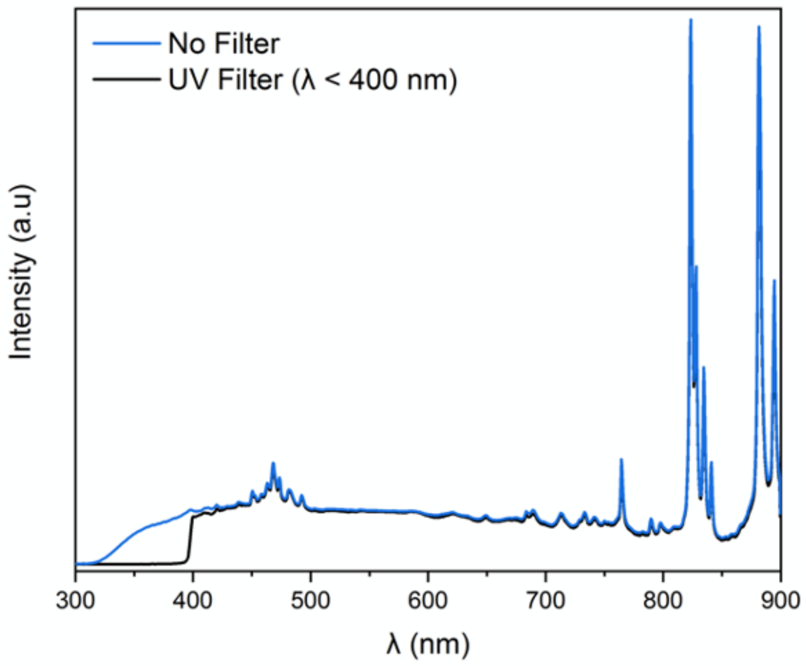

Figure S13. Xenon arc lamp emission spectra, (300W, LOT Quantum Design), equipped with a water filter.

Table S3. Photocatalytic evolution rates of HCP-1, HCP-2, HCP-3, and TiO_2 P25 and control experiments

Sample	UV-vis (Sacrificial H_2)		UV-vis (Sacrificial H_2O)		Visible (Sacrificial H_2O)	
	CO^*	CH_4^*	CO^*	CH_4^*	CO^*	CH_4^*
HCP-1	3.7 ± 0.1	0	8.6 ± 0.1	0.4 ± 0.1	4.2	0.1
HCP-2	1.6 ± 0.3	0.1 ± 0.1	4.6 ± 0.5	0.4 ± 0.2	1	0
HCP-3	6.3 ± 0.2	0.5 ± 0.1	15.6 ± 0.9	0.4 ± 0.1	5.2	0.1
P25 (TiO_2)	6.4 ± 0.4	0.3 ± 0.2	14.7 ± 0.9	0.3 ± 0.1	0.69	0.2
HCP-1[a] "dry condition"	-	-	2.87	-	-	-
HCP-2[a] "dry condition"	-	-	1.67	-	-	-
HCP-3[a] "dry condition"	-	-	6.0	-	-	-
N_2/H_2O, no catalyst	-	-	0	-	-	-
N_2/H_2O [b]	-	-	3.58 (23% [c])	-	-	-
N_2/H_2O repeat [b,c]	-	-	3.78 (24% [c])	-	-	-
N_2/H_2O [b]	-	-	-	-	0.82 (15% [c])	-
N_2/H_2O repeat [b,c]	-	-	-	-	1.06 (20% [c])	-
N_2/H_2 [b]	0.74 (12% [c])	-	-	-	-	-
N_2/H_2 repeat [b,c]	0.61 (10% [c])	-	-	-	-	-
CO_2/ H_2O, no light [b]	-	-	0.16 (1% [c])	-	-	-
CO_2/ H_2O, no catalyst	-	-	10 ppm	-	-	-

* All data are given in $\mu mol.g^{-1}.h^{-1}$

[a] samples were exposed to a humid environment (>99% humidity) overnight before photocatalysis. The photoreactor was purged and filled with only dry CO_2 before irradiation.

[b] tests were performed on HCP-3, the leading material of this study

[c] repeat samples were done on the same sample as the first one, without opening the photo-reactor

[d] percentage compared to the total activity of HCP-3

Table S4. Photocatalytic evolution rates of HCP-1, HCP-2, HCP-3 in μmol.cm^{-2}·h^{-1}

Sample	UV-vis (Sacrificial H_2O)	Visible (Sacrificial H_2O)
	CO*	CO*
HCP-1	8.14*10^{-3}	4.68*10^{-3}
HCP-2	6.93*10^{-3}	2.18*10^{-3}
HCP-3	17.96 *10^{-3}	7.23*10^{-3}

* All data are given in μmol.cm^{-2}·h^{-1}

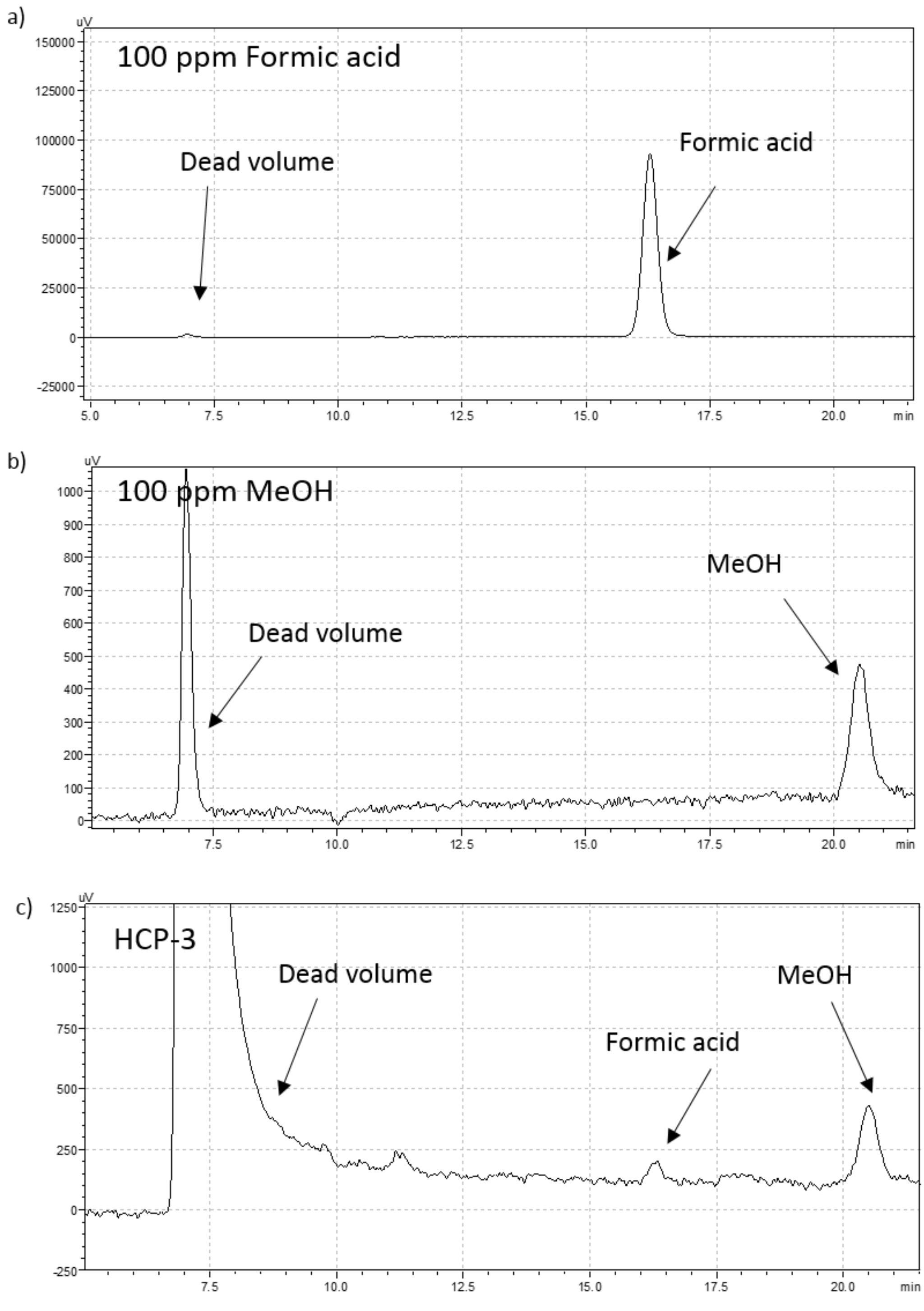

`

Figure S14. HPLC measurements of a) a 100 ppm solution of formic acid, b) a 100 ppm solution of MeOH and c) an aqueous solution derived from washing with deionised water HCP-3 after 3 h UV-vis irradiation under a CO_2 stream and using water as sacrificial agent.

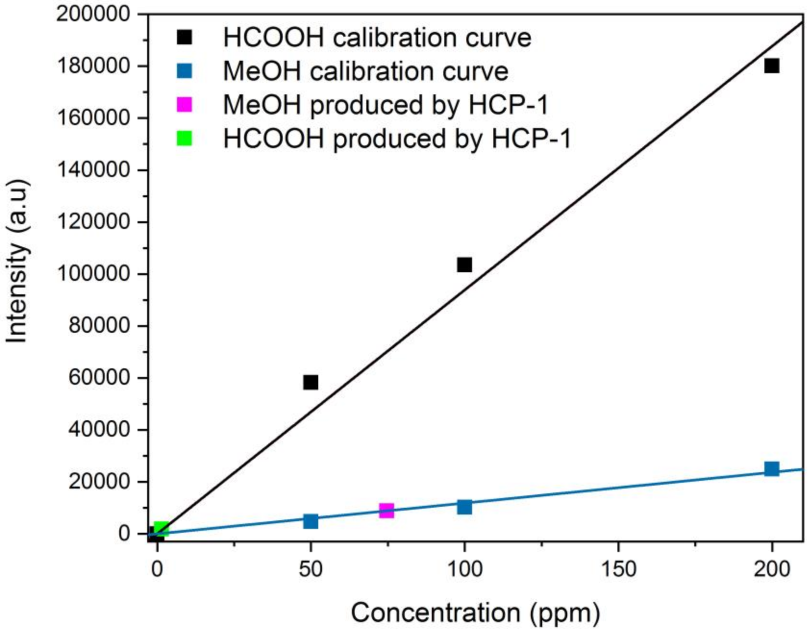

Figure S15. HPLC calibration curves to estimate the concentrations of formic acid and methanol produced during CO_2 photoreduction. The data points obtained when using HCP-1 are indicated on the graphs.

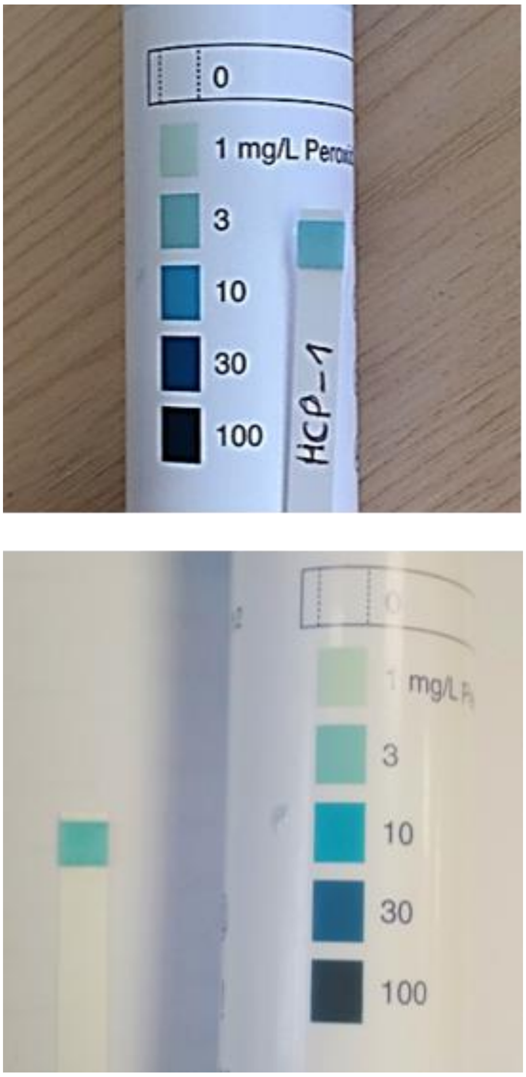

Figure S16. Results of the colorimetric test carried out to detect the presence of H_2O_2 as a result of the photocatalytic process. H_2O_2 was detected for both HCP-1 (top) and HCP-3 (bottom). A concentration of around 3 ppm was detected after 3 h of UV-vis irradiation for HCP-1 and of around 7 ppm for HCP-3.

Quantum efficiency (QE) calculations

The quantum efficiency at a given wavelength λ is defined as:

$$QE = \frac{N_e}{N_p} = \frac{no.of\ reacted\ electrons}{no.of\ absorbed\ photons}\ x100 \qquad \text{(Equation S1)}$$

Based on the stoichiometry of the CO evolution redox reaction:

$$QE = \frac{2N_{CO}}{N_p} = \frac{(2)(no.of\ evolved\ CO\ molecules)}{no.of\ absorbed\ photons}\ x100 \qquad \text{(Equation S2)}$$

For a polychromatic light source, we must consider the total number of absorbed photons across the wavelength range (270 – 900 nm for UV-vis and 400 – 900 nm for visible irradiation) and the corresponding total moles of CO evolved during the irradiation time:

$$QE = \frac{2N_{CO\ (total)}}{N_p} = \frac{(2)(\ total\ no.of\ evolved\ CO\ molecules)}{no.of\ absorbed\ photons}\ \text{x}100 \qquad \text{(Equation S3)}$$

The number of photons absorbed by the photocatalyst at a given wavelength λ is given by:

$$N_{P,absorbed} = \frac{(P_{abs})(\lambda)(t)}{(hc)} \qquad \text{(Equation S4)}$$

where λ denotes the wavelength, P denotes the power of the irradiation source, t denotes the irradiation time, h denotes Planck's constant and c the speed of light.

Equation S4 can be written in terms of the intensity of the irradiation as a function of the irradiation absorbed at wavelength λ, $[I_{abs}(\lambda)]$, and irradiation area (S) as:

$$N_{P,absorbed} = \frac{[I_{abs}(\lambda)](S)(\lambda)(t)}{(hc)} \qquad \text{(Equation S5)}$$

To account for the number of photons absorbed by the photocatalyst at a given wavelength λ, we need to consider the absorbance (Abs) of the material at that wavelength and apply the Beer-Lambert law:

$$Abs(\lambda) = -log\left[\frac{I_F(\lambda)}{I_I(\lambda)}\right] \quad \text{(Equation S6)}$$

where $I_I(\lambda)$ denotes the intensity emitted from the irradiation source at a given wavelength λ and $I_F(\lambda)$ denotes the intensity after passing through the photocatalyst at the same wavelength λ.

The intensity of irradiation absorbed by the photocatalyst at a given wavelength λ is given by:

$$I_{abs}(\lambda) = I_I(\lambda) - I_F(\lambda) \quad \text{(Equation S7)}$$

which, using equation (S6), can be written as:

$$I_{abs}(\lambda) = I_I(\lambda) - I_I(\lambda)10^{-Abs(\lambda)} = I_I(\lambda)\left[1 - 10^{-Abs(\lambda)}\right] \quad \text{(Equation S8)}$$

Substituting equation (S8) into equation (S5) gives

$$N_{P,absorbed} = \frac{I_I(\lambda)\left[1-10^{-Abs(\lambda)}\right](S)(\lambda)(t)}{(hc)} \quad \text{(Equation S9)}$$

The total number of photons absorbed by the photocatalyst across the entire wavelength range of the irradiation source is then given by:

$$N_{P,absorbed\,(total)} = \sum_{\lambda_{start}}^{\lambda_{end}} \frac{I_I(\lambda)\left[1-10^{-Abs(\lambda)}\right](S)(\lambda)(t)}{(hc)} \quad \text{(Equation S10)}$$

$$N_{P,absorbed\,(total)} = \frac{(S)(t)}{(hc)} \sum_{\lambda_{start}}^{\lambda_{end}} I_I(\lambda)\left[1 - 10^{-Abs(\lambda)}\right](\lambda) \quad \text{(Equation S11)}$$

The total number of evolved CO molecules during a given reaction cycle can be written as

$$N_{CO(total)} = (N_a)(n_{CO,total}) \quad \text{(Equation S12)}$$

where N_a denotes Avagadro's constant and $n_{CO,total}$ denotes the total moles of CO evolved during the irradiation time.

Substituting equations (S11) and (S12) to (S3) gives us the final expression for the quantum efficiency for CO evolution:

$$QE = \frac{(2)\, N_{CO\,(total)}}{N_{\mathrm{P}}} = \frac{(2)(N_a)(n_{CO,total})}{\frac{(S)(t)}{(hc)}\sum_{\lambda_{start}}^{\lambda_{end}} I_I(\lambda)\left[1-10^{-abs(\lambda)}\right](\lambda)} \text{ x100} \qquad \text{(Equation S13)}$$

Table S5. Quantum efficiency calculation of HCP-3

Sample	UV-vis irradiation (sacrificial H_2O)	Visible irradiation (sacrificial H_2O)
HCP-3	0.002 %	0.0005 %

*The quantum efficiency was calculated from a polychromatic light source. The total number of absorbed photons across the wavelength range (270 – 900 nm for UV-Vis and 400 – 900 nm for visible irradiation) was taken into consideration.

Table S6. Apparent quantum yields (AQY) or quantum efficiencies (QE) and production rates of HCP-3 and a selection of organic photocatalysts.

Type of photocatalysts	Phase	Irradiation conditions	Production rate (umol.g^{-1}.h^{-1})	AQY/QE	Ref
Triphenylamine based conjugated microporous polymers	Gas	300 W Xe lamp, >420 nm	37	0.19% (420 nm)	4
Conjugated polymer (PEosinY-1)	Gas	300 W Xe lamp, >420 nm	33	-	5
Triazine based 2D COF	Gas	300 W high pressure Xe lamp, >420 nm	103	0.010% (full spectrum, QE)	6
Boron-doped carbon nitride	Gas	500 W Xe arc lamp, Uv-vis	1.23	-	7
Oxygen-doped boron nitride	Gas	300 W Xe lamp, UV-vis	12.5	-	8
HCP-3 (This study)	Gas	300 W Xe lamp, UV-vis	15.6	0.002% (full spectrum, QE)	-
HCP-3 (This study)	Gas	300 W Xe lamp, >420nm	5.2	0.0005% (full spectrum, QE)	-

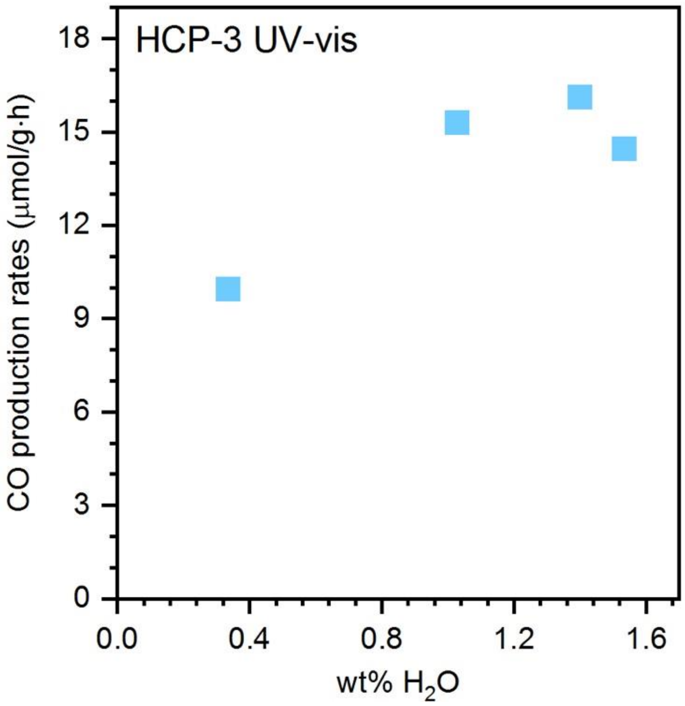

Figure S17. CO production rates of HCP-3 under UV-vis irradiation using different water vapour contents.

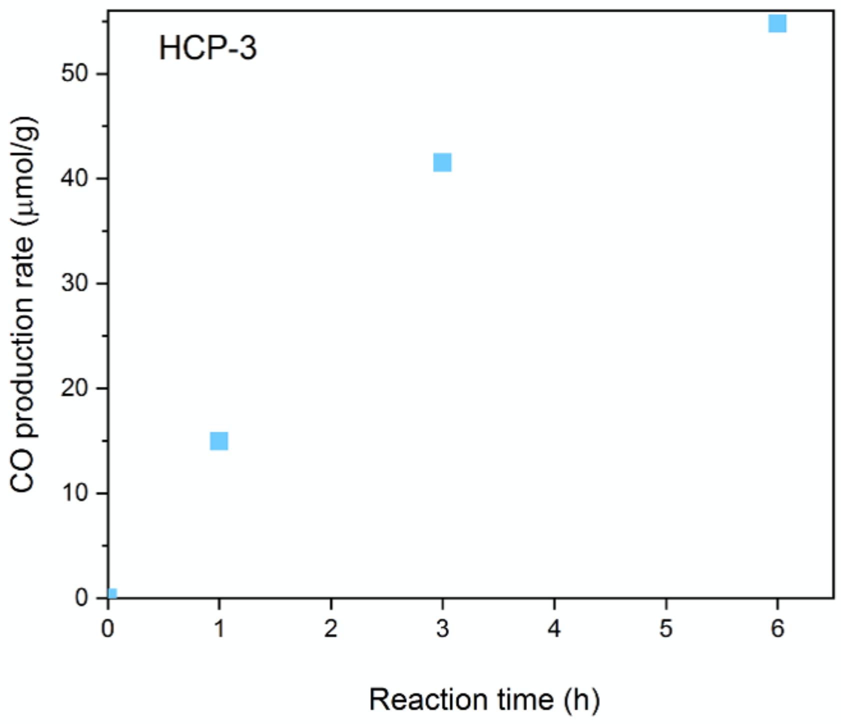

Figure S18. Photocatalytic CO evolution from HCP-3 under UV-vis irradiation as a function of time.

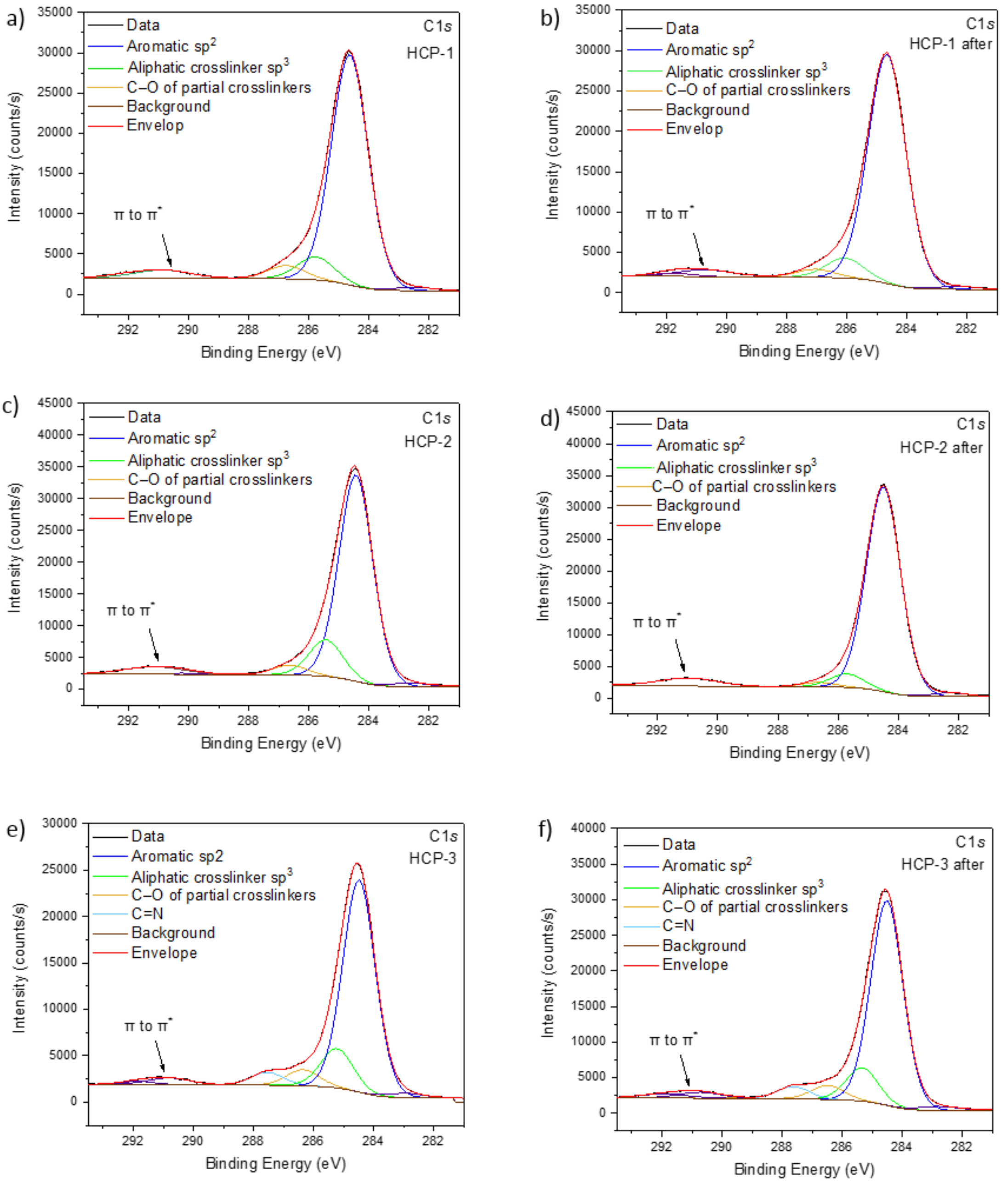

Figure S19. Comparison of C1*s* core level spectra before and after the CO_2 photoreduction reaction of HCP-1, HCP-2, and HCP-3.

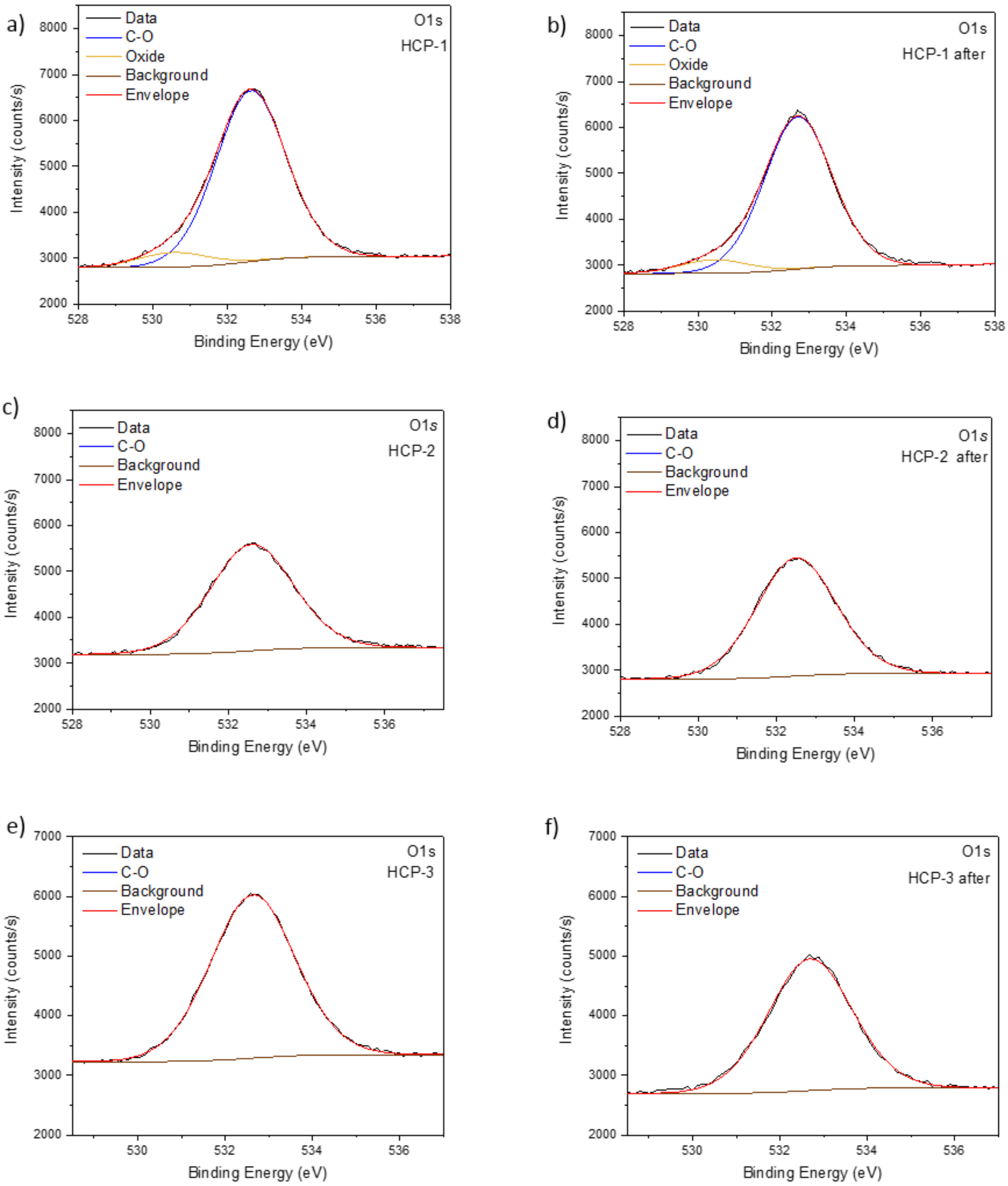

Figure S20. Comparison of O1*s* core level spectra before and after the CO_2 photoreduction reaction of HCP-1, HCP-2, and HCP-3.

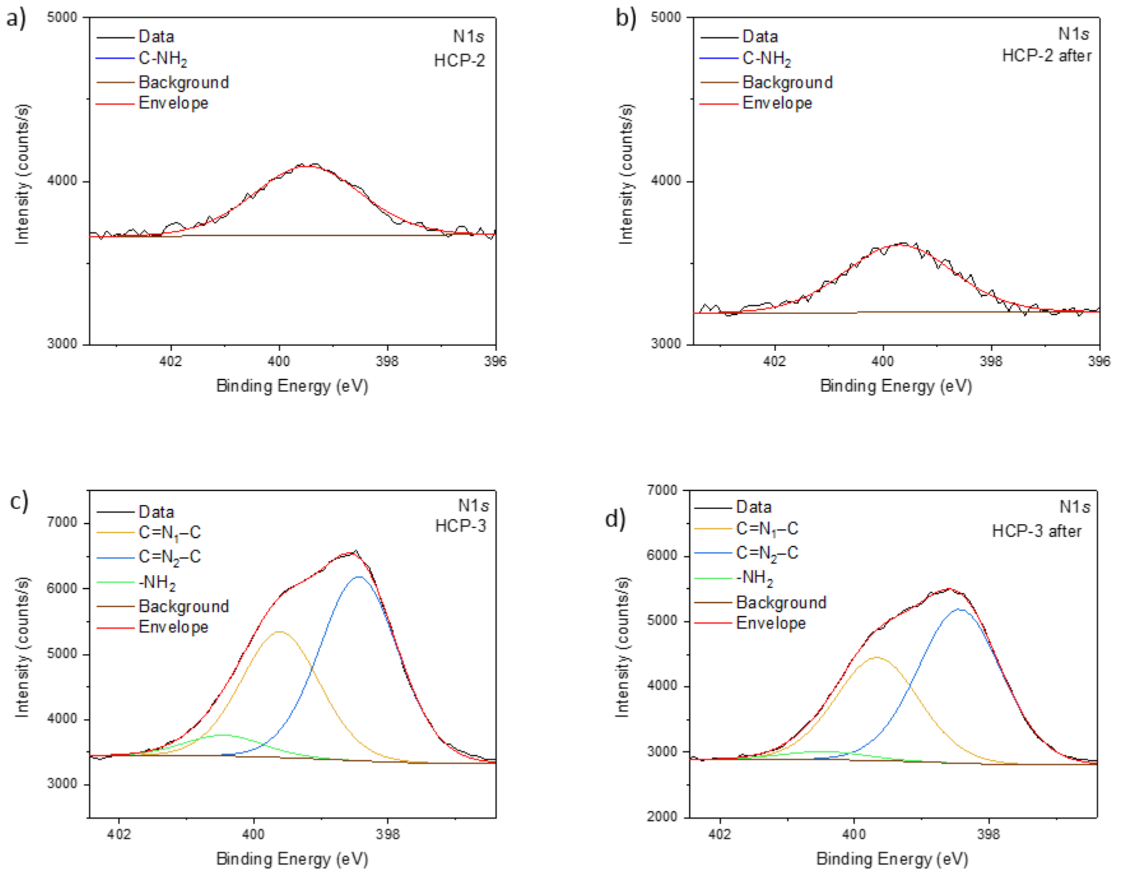

Figure S21. Comparison of N1*s* core level spectra before and after the CO_2 photoreduction reaction of HCP-2 and HCP-3.

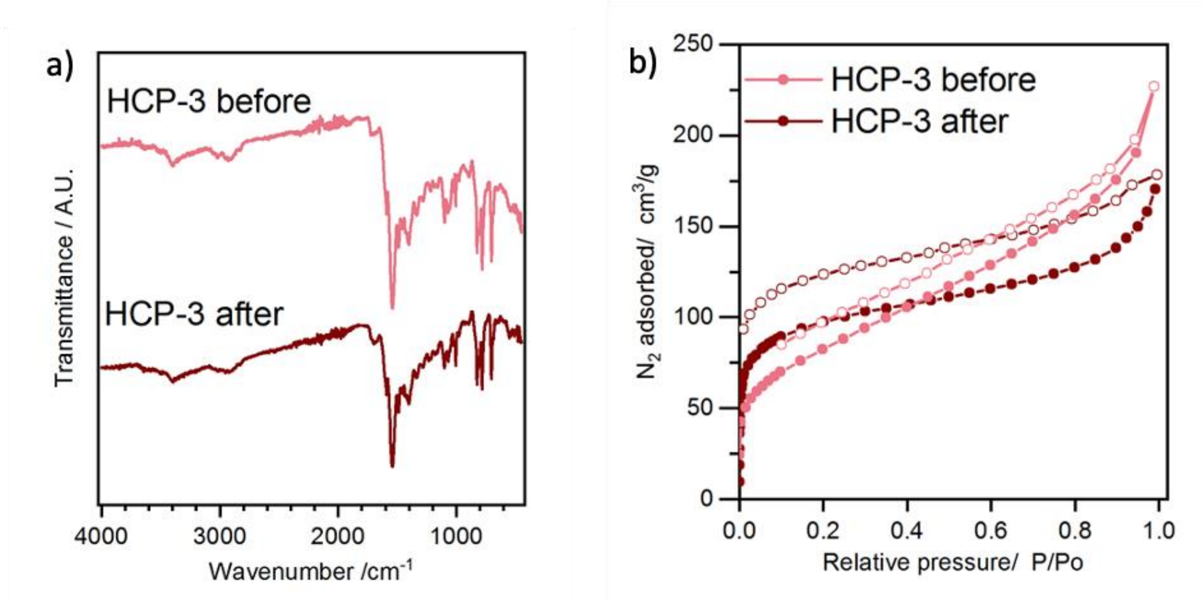

Figure S22. a) FTIR, and b) N_2 adsorption isotherms at 77 K of HCP-3 before and after the CO_2 photoreduction reaction.

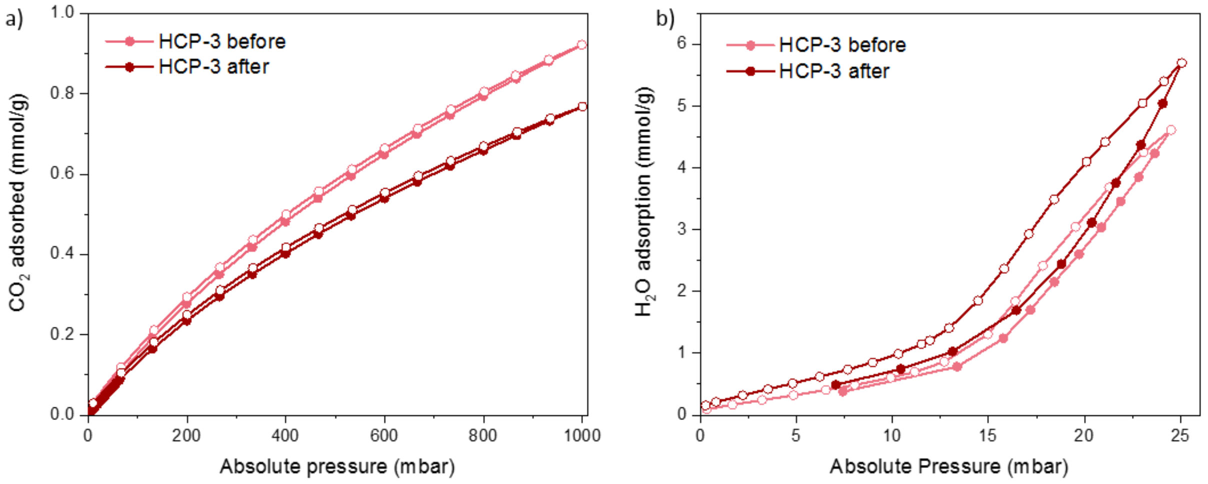

Figure S23. a) CO_2 adsorption isotherms and b) H_2O adsorption isotherms at 298 K of HCP-3 before and after the CO_2 photoreduction reaction.

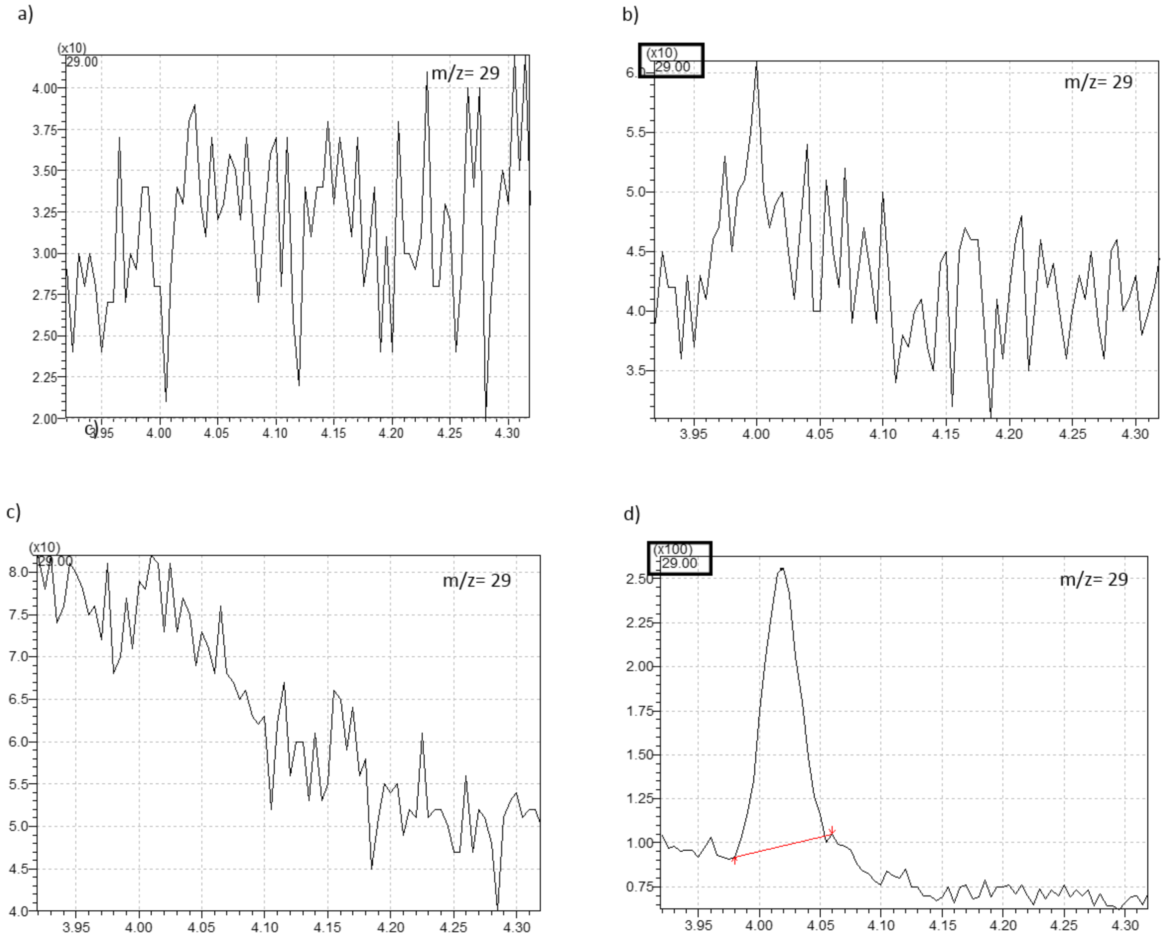

Figure S24. $^{12}CO_2$ and isotopic $^{13}CO_2$ measurements. Mass spectrum chromatogram of HCP-3 illustrating the ^{13}CO (m/z = 29) peak observed with $^{12}CO_2$ after a) 0 min and b) 4h UV-vis irradiation, and the ^{13}CO (m/z = 29) peak observed with $^{13}CO_2$ after c) 0 min irradiation and d) 4h UV-vis irradiation

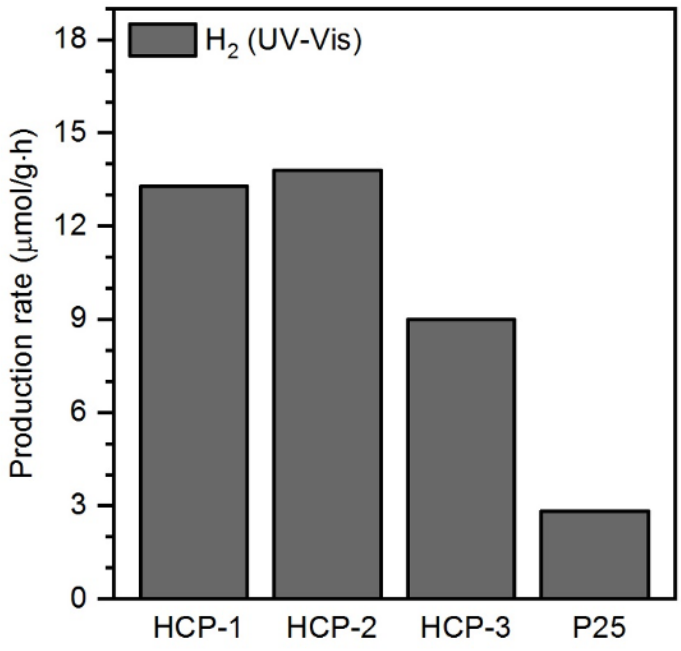

Figure S25. H_2 production rates of HCPs under UV-vis irradiation using H_2O as sacrificial agent.

3. References

1. S. Brunauer, P. H. Emmett and E. Teller, *Journal of the American chemical society*, 1938, **60**, 309-319.
2. S. Chen and R. Yang, *Langmuir*, 1994, **10**, 4244-4249.
3. A. Crake, K. C. Christoforidis, A. Gregg, B. Moss, A. Kafizas and C. Petit, *Small*, 2019, **15**.
4. C. H. Dai, L. X. Zhong, X. Z. Gong, L. Zeng, C. Xue, S. Z. Li and B. Liu, *Green Chem*, 2019, **21**, 6606-6610.
5. X. X. Yu, Z. Z. Yang, B. Qiu, S. E. Guo, P. Yang, B. Yu, H. Y. Zhang, Y. F. Zhao, X. Z. Yang, B. X. Han and Z. M. Liu, *Angew Chem Int Edit*, 2019, **58**, 632-636.
6. K. Lei, D. Wang, L. Q. Ye, M. P. Kou, Y. Deng, Z. Y. Ma, L. Wang and Y. Kong, *Chemsuschem*, 2020, **13**, 1725-1729.
7. N. Ojha, A. Bajpai and S. Kumar, *Catal Sci Technol*, 2020, **10**, 2663-2680.
8. Y. H. Cao, R. Y. Zhang, T. L. Zhou, S. M. Jin, J. D. Huang, L. Q. Ye, Z. A. Huang, F. Wang and Y. Zhou, *Acs Appl Mater Inter*, 2020, **12**, 9935-9943
